# The Power of Universal Contextualized Protein Embeddings in Cross-species Protein Function Prediction

**DOI:** 10.1177/11769343211062608

**Published:** 2021-12-03

**Authors:** Irene van den Bent, Stavros Makrodimitris, Marcel Reinders

**Affiliations:** 1 Delft Bioinformatics Lab, Delft University of Technology, Delft, the Netherlands; 2 Keygene N.V., Wageningen, the Netherlands

**Keywords:** Protein function prediction, protein language models, protein embedding, transfer learning, annotating evolutionary distant proteins

## Abstract

Computationally annotating proteins with a molecular function is a difficult problem that is made even harder due to the limited amount of available labeled protein training data. Unsupervised protein embeddings partly circumvent this limitation by learning a universal protein representation from many unlabeled sequences. Such embeddings incorporate contextual information of amino acids, thereby modeling the underlying principles of protein sequences insensitive to the context of species. We used an existing pre-trained protein embedding method and subjected its molecular function prediction performance to detailed characterization, first to advance the understanding of protein language models, and second to determine areas of improvement. Then, we applied the model in a transfer learning task by training a function predictor based on the embeddings of annotated protein sequences of one training species and making predictions on the proteins of several test species with varying evolutionary distance. We show that this approach successfully generalizes knowledge about protein function from one eukaryotic species to various other species, outperforming both an alignment-based and a supervised-learning-based baseline. This implies that such a method could be effective for molecular function prediction in inadequately annotated species from understudied taxonomic kingdoms.

Proteins are diverse molecules that perform many different functions in cells, ranging from catalyzing chemical reactions to functioning as mere structural components.^1-3^ These functions are generally described in terms of functional Gene Ontology (GO) annotations. GO annotations, also known as GO terms, are statements about the molecular function of the protein, cellular localization of the protein or the biological process it supports.^4^ This knowledge on protein function has come to play a central role in our daily lives, fueling the field of synthetic biology and thereby solving problems in medicine, manufacturing, and agriculture.^5-7^

To date, however, most GO annotations linked to proteins are shallow and incomplete.^8,9^ Additionally, as increasingly more protein sequences are characterized by high-throughput wet-lab experiments, they often remain without any functional annotation.^10,11^ Especially in certain taxonomic kingdoms such as the Plantae, Protozoa, and Chromista, very few species have been thoroughly studied and the quality of available annotations is substandard.^12^

Extensive wet-lab experiments remain the most accurate tools to annotate proteins but are time-consuming, expensive and some proteins cannot be studied at all due to technical limitations.^13^ In response, there have been numerous attempts to functionally annotate proteins using automated, fast and scalable bioinformatics tools.^14,15^ Early approaches like BLAST often rely on homology relationships to identify conserved protein sequences, transferring the functional annotation between them, as conserved sequence implies conserved function.^16,17^ These kinds of approaches quickly drop in predictive power for divergent proteins and, therefore, new generation approaches often integrate numerous types of protein data with potent computational tools like neural networks. These types of protein data include sequence motifs, structural motifs, co-expression data and protein-protein interactions usually extracted from (the combination of) amino acid sequences, 3D structures and high-throughput techniques.^14,18,19^ A prime example of such a method is GOLabeler,^20^ which uses a powerful data integration algorithm to combine predictions from multiple sources, reliably outperforming all other methods in molecular function prediction in the CAFA3 challenge.^21^ Whereas the new generation techniques usually outperform the established BLAST baseline method, they also require vast amounts of protein data which is not always comprehensive. Therefore, the most recent approaches often turn to automatic representation learning by which a complex model (often a neural network) learns some abstract features of a protein sequence that contains useful information for a consequent computational function prediction task.^22-24^

Recently, we demonstrated that features generated from the pre-trained protein language model Seqvec^25^ significantly outperform methods that learn sequence features in a supervised manner, even when coupled with a simple linear classifier.^24^ The advantage of language models is that they are trained in an unsupervised fashion, by training to predict each amino acid in a protein sequence given its “context,” that is neighboring amino acids.^25-29^ This means that they can be trained on all available protein sequences and not only the annotated ones. By leveraging this wealth of data, they can learn general properties of amino acids, such as polarity and secondary structure, that are very useful for downstream prediction tasks.^25^ Consequently, deep supervised methods can be build upon these learned embeddings so that their learning can be done with a relatively small number of proteins available for training.^24^

The SeqVec model, whose architecture is shown in Figure 1a, produces a 1024-dimensional embedding for every amino acid in the protein sequence (Figure 1b). Protein-level embeddings are obtained by calculating the component-wise mean over the sequence of amino acid-level embeddings (Figure 1c). This results in a final 1024-dimensional protein-level embedding independent of the protein sequence length.

**Figure 1.**
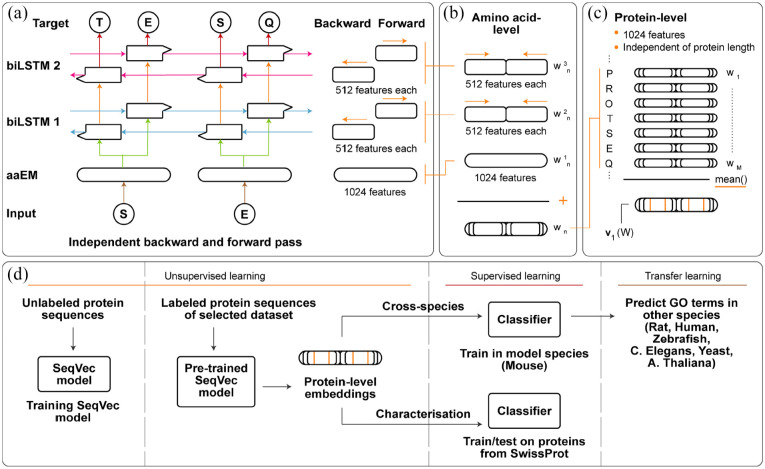
(a) SeqVec model architecture holds 3 layers: an amino acid embedding layer (aaEM), the first bidirectional Long Short Term Memory (biLSTM 1) and the second biLSTM (biLSTM 2). The aaEM maps the input amino acid onto a latent 1024-dimensional space. Both biLSTM’s have a separate forward and backward pass to incorporate information on the previous or following amino acids and map this information onto 512-dimensional spaces. Based on the biLSTM 2 embeddings, the model predicts the next or previous amino acid in the sequence. Weights between the forward and backward pass are shared, represented here as arrows with the same color. (b) During inference, the embeddings of both biLSTM layers are concatenated to 1024-dimensional embeddings. Using the standard approach from Heinzinger et al,^25^ a final amino acid-level embedding is obtained by summing the 1024-dimensional embeddings $w_{n}^{1},w_{n}^{2}$ $w_{n}^{3}$ resulting in a 1024-dimensional contextualized embedding $w_{n}$ for every amino acid n in the protein sequence. (c) Protein-level embeddings are obtained by calculating the component-wise arithmetic mean of the sequence of amino acid embeddings $\left(w_{1},\ldots ,w_{n}\right)$ resulting in 1024-dimensional protein-level embedding $v_{1}\left(W\right)$ independent of protein length. (d) Overview of approaches taken in this study. The pre-trained SeqVec model is used to embed proteins in a 1024-dimensional space. In the cross-species experiments, we train a molecular function predictor on a “central’ species and evaluate its performance on proteins of other species. In our characterization experiments, we use the embeddings to get a deeper understanding of SeqVec-based molecular function prediction performance.

In this paper, we build upon previous work^24,25^ and use the SeqVec embeddings to examine the reliability of predicting functions between proteins of evolutionary distant species. Till date, making predictions over large evolutionary distances is difficult as the function of a protein is determined by the context of its species.^15^ If SeqVec can truly learn the underlying principles of protein sequences, we expect SeqVec-based protein function prediction to be much less sensitive to this limitation as SeqVec then will produce universal embeddings independent of the context of species. If proven effective, this particular cross-species approach could be especially useful for understudied evolutionary kingdoms (eg, Plantae) to readily generalize knowledge on protein function.

The concept of cross-species prediction has been previously touched upon when Jensen et al^30^ showed that knowledge on protein “cellular roles,” which are much broader statements about protein functions than molecular functions, can be generalized from one eukaryotic species to another eukaryotic species. They made their predictions using the ProtFun method that uses as input hand-crafted features, such as post-translational and localization aspects of the protein. We extend this work by (i) using the more powerful SeqVec model and (ii) predicting more specific molecular functions instead of broad cellular roles of proteins. We train a SeqVec-based molecular function prediction model on the annotated proteins from one training species (Mouse) and assess the performance of the trained model on the proteins of other, evolutionary related and distant species (Figure 1d). As proof of principle, we use the data from well-annotated eukaryotic species to aid performance evaluation which typically relies on comparing predicted functions to the true functions of proteins.

In summary, we demonstrate the effectiveness and reliability of this data-undemanding approach by successfully transferring knowledge on protein function between the training species and various other eukaryotic species. Thereby, we present an innovative method for molecular function prediction in inadequately annotated species from understudied taxonomic kingdoms.

As language models are relatively novel in the field of protein function prediction, we also submit the performance of SeqVec-based molecular function prediction models to a detailed characterization, to advance the understanding of such models (Figure 1d). In doing so, we uncover a clear relationship between performance and the diversity of the proteins that perform a function in terms of domains and protein families.

## Results Characterization

### Evaluating SeqVec-based molecular function prediction performance

Previously we showed that SeqVec embeddings achieve competitive performance when applied in the task of molecular function prediction.^24^ Here, we have used the same so-called SwissProt dataset (30% maximum-pairwise sequence similarity, 3530 test proteins and 441 GO terms). Although our previous work revealed that a multilayer perceptron (MLP) trained on protein-level embeddings had the best performance, the MLP was also trained in a multilabel fashion. Hence, underlying relations between GO annotations and their abundance in the training set might have influenced the MLP performance in ways we are unsure about. As a Logistic Regression (LR) can easily be trained independently for every GO term (and showed only slightly lower performance than the MLP in our previous work), we use the LR to characterize the performance. Throughout this study, we evaluated the performance in a term-centric (ROCAUC score) and protein-centric (F1 score) way as proposed and described in detail by Radivojac et al.^15^

**Figure 2.**
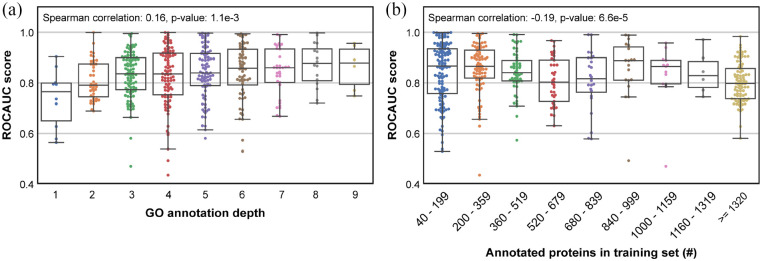
Term-centric performance (ROCAUC) per GO term of a LR classifier trained using SeqVec protein-level embeddings on the SwissProt dataset with at most 30% pairwise sequence identity in relation to (a) depth of the GO term and (b) the number of annotated proteins in the training set for the GO term. The Spearman correlations and corresponding *P*-values are shown in the respective plots. The box-whiskers plots show the interquartile range (IQR) with a box and the median as a bar across the box. Whiskers denote the range equal to 1.5 times the IQR.

First, we observed similar performance values as our previous work with an average ROCAUC score of 0.832 (95% confidence interval [0.827-0.837]) and an average F1 score 0.479 (95% confidence interval [0.470-0.484]). Additionally, the coverage was 0.998, indicating that for almost all proteins in the test set at least one molecular function was predicted. We observed a weak non-linear positive correlation between GO term depth and term-centric performance (Spearman correlation: 0.16, *P*-value: 1.1e-3) (Figure 2a). For clarity, depth was defined as the length of the longest possible path to a GO term from the root term in the GO hierarchy. The number of proteins in the training set and term-centric performance showed a weak non-linear negative correlation (Spearman correlation: −0.19, *P*-value: 6.6e-5) (Figure 2b). However, even between terms with the same depth or number of training proteins, we observed a large spread in the term-centric performance. We also note that as a result of the GO hierarchy, the depth and number of training proteins were not independent parameters, that is terms closer to the root (ie, with low depth) usually have more annotated proteins (Spearman correlation: −0.34, *P*-value: 1.55e-13).

### Protein-centric performance correlates positively with protein length

To test whether protein characteristics could be underlying the differences in term-centric performance, we characterized the protein-centric performance to protein length and the number of protein annotations. Again, these parameters were not independent as in eukaryotes the protein size is positively correlated with more extended multifunctional proteins.^31,32^ However, in the SwissProt dataset, we observed only a mild correlation which we attributed to the lack of true annotations for many proteins (Spearman correlation: 0.14, *P*-value: 1.55e-13).^8,9^

**Figure 3.**
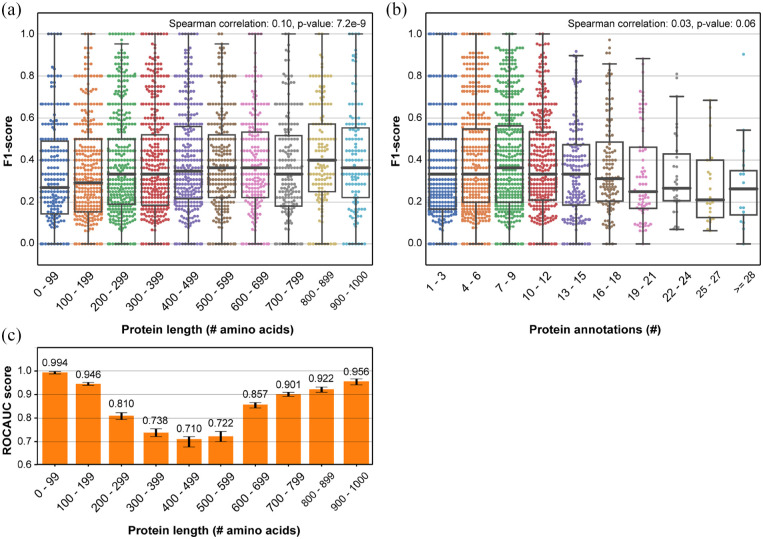
Protein-centric performance (F1) per protein of the LR classifier trained using baseline SeqVec protein-level embeddings on the SwissProt dataset in relation to (a) protein sequence length and (b) the number of protein annotations. The LR was trained to predict GO terms. The Spearman correlations and corresponding *P*-values between protein-centric performance and protein length or number of protein annotations are shown. The box-whiskers plots show the interquartile range (IQR) with a box and the median as a bar across the box. Whiskers denote the range equal to 1.5 times the IQR. (c) Term-centric performance (ROCAUC) of the LR classifier trained using baseline SeqVec protein-level embeddings on the SwissProt dataset. The LR was trained to predict protein length encoded by one-hot encoding in the same intervals as in (a). Errorbars denote 95% confidence estimated using 100 bootstraps.

We observed a weak non-linear positive correlation between protein length and protein-centric performance (Spearman coefficient: 0.10, *P*-value: 7.2e-9) (Figure 3a). The slightly increased protein-centric performance for longer proteins was mainly the result of improved precision for longer proteins with recall remaining similar for all protein lengths (Figure S.1A and B). For an increased number of protein annotations we observed a slightly decreased protein-centric performance, although testing for a correlation resulted in no statistical significant finding (Spearman coefficient: 0.03, *P*-value: 0.06) (Figure 3b). Here, precision increased for more annotations while recall decreased (Figure S.1C and D).

Overall, the difference in protein-centric performance between long and short proteins was small. Therefore, even if some GO terms with the same depth or number of training proteins had large differences in the average length of their annotated proteins, our evaluated protein characteristics seemed an unlikely source of the differences in term-centric performance. However, in theory, taking the mean over a larger number of amino acid-level embeddings discards more information as it results in an average closer to the population mean, whereas taking the mean over less amino acid-level embeddings should give an average closer to the sample mean.^33^ Hence, we expected a lower protein-centric performance for longer proteins. This was not the case, hinting that SeqVec embeddings likely model some protein characteristic that countered the expected decrease in performance for longer proteins.

### Protein-level embeddings effectively model protein length

To explain the observed positive correlation, we hypothesized that the protein length is somehow encoded in SeqVec embeddings. Specifically, as long proteins are relatively scarce, they might be easier to predict by having similar embeddings as other long protein in the training set with similar function. To test our hypothesis, we trained a LR classifier on the protein-level embeddings to predict protein length. We binned the protein length similar as in Figure 3a and modeled these bins by one-hot encodings.

Indeed, we observed that protein length was modeled by the protein-level embeddings, as reflected by an average ROCAUC score of 0.856 taken over all protein length intervals of the LR classifier (Figure 3c). Specifically, the performance was high for very short or very long proteins, and moderate for proteins with a more average length. Overall, this finding indicated that embeddings still capture relevant information on protein size, even though they are obtained by taking the mean over amino acid embeddings.

### Term-centric performance correlates positively with an increased domain, family, and superfamily similarity between proteins

As an alternative approach to explain the differences in term-centric performance, we hypothesized that differences in similarity between proteins annotated with certain GO terms might be the underlying cause. For example, proteins with the same domain tend to perform similar molecular functions. SeqVec embeddings might not be able to capture these domains. In that case, GO terms with proteins that are more structurally dissimilar but functionally similar (eg, same domains) should have a lower term-centric performance.^34,35^ To test this, we retrieved the following annotations from the InterPro database: (i) protein domains (ie, structurally conserved functional units) (ii) protein families (ie, evolutionarily related proteins with similar three-dimensional shapes), and (iii) protein superfamilies (ie, structurally/mechanistically related proteins not necessarily evolutionary related). As these annotations were sparse, we were unable to retrieve annotations for all test proteins.

**Figure 4.**
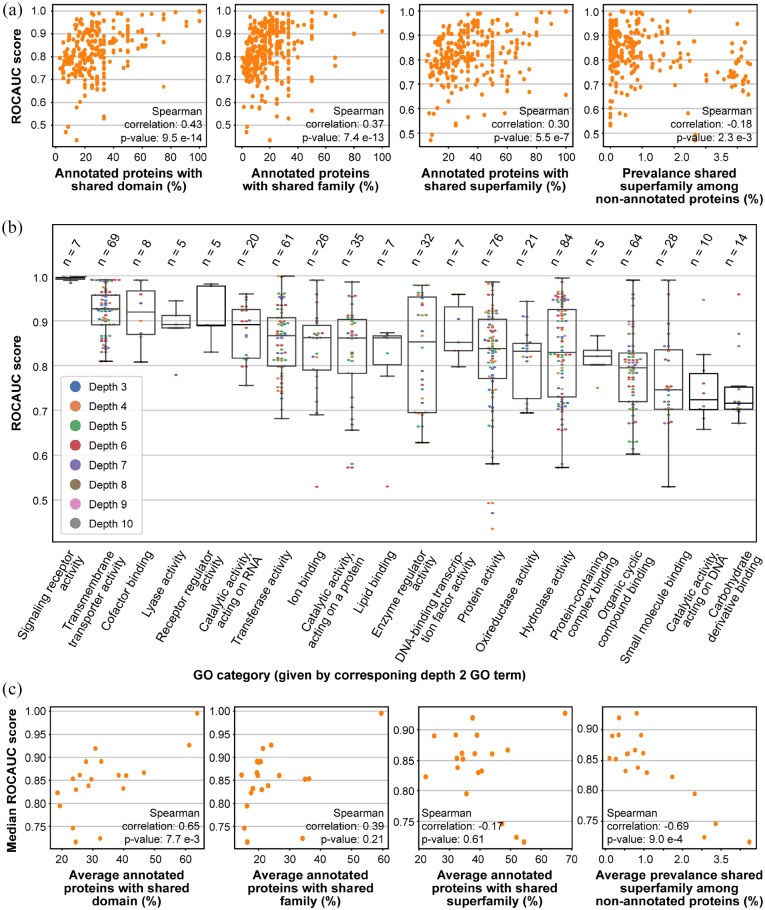
(a) ROCAUC per GO term of the SeqVec-based LR classifier in relation to the domain/family/superfamily similarity of proteins annotated with that term. From left to right: the performance in relation to the percentage of annotated proteins with a shared domain, family or superfamily. On the far right: performance in relation to the prevalence of the shared superfamily among the remaining non-annotated proteins. (b) ROCAUC per GO term of the same classifier in relation to GO category. Child GO terms in each GO category are shown as dots with color indicating their depth. The number of child terms per category is presented at the top. The box-whiskers plots show the standard IQR, median and 1.5 × IQR. (c) As in A, but terms are grouped per GO category and the median ROCAUC is shown for each category.

To quantify the domain, family or superfamily similarity between proteins annotated with a certain GO term, we evaluated what percentage of them shared a domain, family or superfamily annotation. Note that these annotations are not independent quantities (Spearman correlations; domains-families: 0.68, *P*-value: 1.6e-29; domains-superfamilies: 0.75, *P*-value: 5.2e-42; families-superfamilies: 0.49, *P*-value: 3.3e-14). We observed moderate non-linear positive correlations between the percentage of proteins sharing a domain, family or superfamily and term-centric performance (Spearman correlations: 0.43, *P*-value: 9.5e-14; 0.37, *P*-value: 7.4e-13; 0.30, *P*-value: 5.5e-7, respectively) (Figure 4a). If we control for the confounding factor of differences in class imbalance across terms, we still find that performance can be predicted by InterPro annotation similarities (Supplemental Material subsection 1.1). This indicates that indeed the term-centric performance for GO terms with many annotated evolutionary related proteins is generally better.

Next, we hypothesized that SeqVec’s ability to model such similarities might be compromised when a protein is annotated with multiple domains, families or superfamilies as more information needs to be captured in the same 1024-dimensional embedding. However, we observed no statistically significant correlations between the average number of domains, families or superfamilies per annotated protein and term-centric performance, indicating that SeqVec embeddings efficiently modeled multiple functionalities of proteins (Figure S.2A).

Finally, as we still observed significant differences in term-centric performance between GO terms with a low percentage of proteins sharing a domain, family or superfamily, we hypothesized that a higher prevalence of the shared domain, family or superfamily among the remaining test proteins could lower performance as they might be predicted as false positives. Indeed, we observed a weak non-linear negative correlation between the prevalence of the shared superfamily for a certain GO term among the remaining test proteins and the term-centric performance (Spearman correlation: −0.18, *P*-value: 2.3e-3) (Figure 4a). We did not observe a statistically significant correlation between the prevalence of the most shared domain or family and the term-centric performance, possibly due to the generally low prevalence of the most shared domain or family in the remaining population (Figure S.2B).

Overall, these correlations hint that some molecular functions are being executed by a wider spectrum of protein families, thereby lowering the term-centric performance of SeqVec-based molecular function prediction.

### High term-centric performance related to specific molecular functions carried out by similar proteins

To identify the molecular functions with many similar annotated proteins, we created so-called “GO categories.” First, we selected all GO terms in the SwissProt dataset with depth two. Next, from this selection, we selected terms with at least 5 child terms. This resulted in twenty “GO categories” indicated by their depth 2 GO term, thereby indicative of certain types of molecular functions.

We ordered the GO categories based on their median term-centric performance and observed large differences in their term-centric median performance and the spread in performance (Figure 4b). Notably outstanding was the GO category of “signaling receptor activity” with a median performance of ∼0.98 and almost no spread. To confirm that the molecular functions of the best performing GO categories were executed by similar proteins, we related the performance of each GO category to the 4 significant correlations on the similarity measures mentioned in the previous section (see Table S.3). As expected, we observed a strong positive correlation between the average percentage of shared domains among the annotated proteins and the median term-centric performance of the GO category (Spearman correlation: 0.65, *P*-value: 7.7e-3) (Figure 4c). Additionally, we observed a strong negative correlation between the average prevalence of the shared superfamily among the remaining proteins and the median term-centric performance of the GO category, indicating that proteins with more common superfamilies can generally be predicted worse (Spearman correlation: −0.69, *P*-value: 9.0e-4). We did not observe a statistically significant correlation between the average percentage of shared families/superfamilies among the annotated proteins and the median term-centric performance of the GO category.

These results, which also hold after controlling for term frequency (Supplementary Material subsection 1.1), suggest that differences in term-centric performance for SeqVec-based molecular function prediction models mainly stem from differences in the divergence between proteins executing the same molecular functions.

## Results Cross-species Function Prediction

### Model species selection

**Figure 5.**
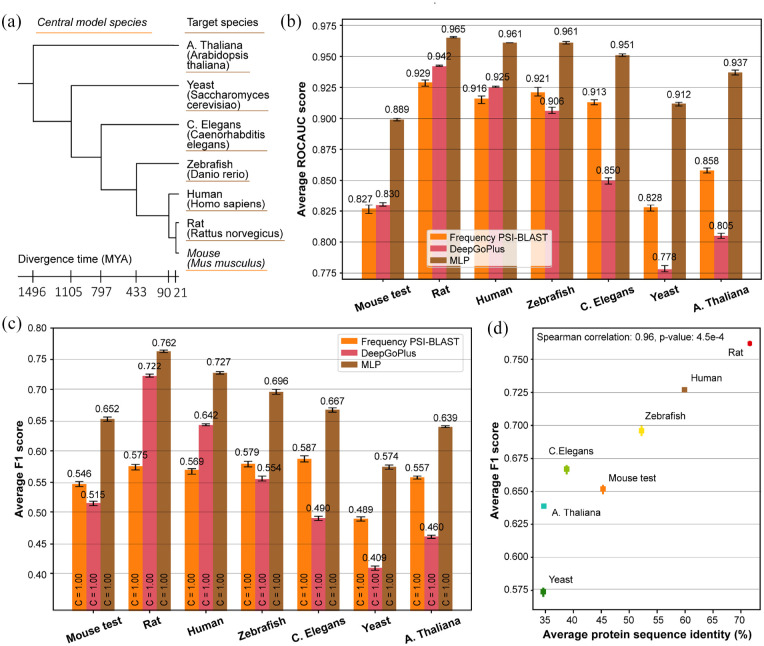
(a) Phylogenetic tree showing evolutionary relation and divergence time between the training species Mouse and the other test species. Tree produced via the PhyloT tool for phylogenetic tree visualization and divergence times retrieved using the TimeTree tool.^37,38^ (b) Average term-centric ROCAUC over all the GO terms and (c) average protein-centric F1 over all the proteins per species for the MLP classifier (brown). The MLP was trained to predict GO terms. Performance is compared to baseline Frequency PSI-BLAST (orange) and to DeepGoPlus (pink). In (c) the coverage C is shown inside the bars. (d) Average protein-centric performance (F1) over all the proteins per species of the MLP in relation to the average protein sequence identity to the Mouse training set. Sequence identity was retrieved using the PSI-BLAST top hit of every protein to the Mouse training set. Errorbars denote 95% confidence intervals estimated using 100 bootstraps.

We assessed if knowledge about protein functions learned in one training species could be generalized to other species. To this end, we trained a SeqVec-based molecular function prediction model on the data of one training species and assessed its performance on the data of several test species with varying evolutionary distance. As proof of principle, we considered seven well-annotated species from different evolutionary classes, phyla and even kingdoms: Mus musculus (Mouse), Rattus norvegicus (Rat), Homo sapiens (Human), Danio rerio (Zebrafish), Caenorhabditis elegans (C. elegans), Saccharomyces cerevisiae (Yeast) and Arabidopsis thaliana (A. thaliana) (Table S.4A).^36^ As these species have different genome sizes, they have a different number of testable protein sequences. To quantify how well these proteins represented all the molecular functions in the species, we calculated the coverage of the gene count, that is how many of the protein-coding genes were represented by the proteins (Table S.4A). We selected Mouse as the training species, creating an ’evolutionary staircase’ in which the remaining species had increasing divergence time from Mouse (Figure 5a). To optimally tune and assess a classifier, we split the Mouse data into 8977 mouse training, 1801 mouse validation, and 1790 mouse test proteins.

Besides the different number of test proteins for each species, we note additional differences in the number of GO terms present among the proteins of each species (Table S.4B). In practice, one would want to predict as many molecular functions as possible, but for this feasibility study we note 2 major limitations: (i) we could not test target species GO terms when they were not present among the Mouse training set GO terms (eg, GO terms related to photosynthesis), and (ii) testing all the Mouse training GO terms in the test species could have predicted annotations for some proteins which we would not be able to reliably validate as data on those functions was lacking. Hence, for protein-centric evaluation (F1-score), we evaluated only the GO terms overlapping between the Mouse training set and the datasets of the test species. For term-centric evaluation (ROCAUC score), we evaluated only GO terms overlapping with the mouse training set and with at least 3 annotated test proteins (Table S.4B).

After the GO term selection process, we checked if the selected GO terms for every species were of similar depth as we previously showed a positive correlation between term-centric performance and GO term depth. We observed similar distributions of GO term depth per species, although for at least 1 species the distribution was significantly different (Chi-square test *P*-value: 2.7e-23) (Figure S.3A). This was not the case for the depth distributions of GO terms selected for term-centric evaluation (Chi-square test *P*-value: 0.20) (Figure S.3B). The observed difference in depth distribution for protein-centric evaluation may have a minor influence on differences in performance between test species.

### Protein functions learned in training species are effectively predicted cross-species

We trained an MLP classifier on the embeddings of the Mouse training set. We specifically select the MLP over LR because our interest now lies in best performance. To evaluate protein-centric performance (F1-score), GO term posterior probabilities were converted into predicted binary class labels using a threshold. To mimic a real-case scenario in which no information on the test species is present, we determined this threshold on the mouse validation set and applied it to posterior probabilities for every test species (Figure S.4).

We observed that the MLP outperformed the baseline Frequency PSI-BLAST method and DeepGOPlus in all species for both term-centric and protein-centric evaluation (Figure 5b and c). The absolute performance of the MLP decreased with increasing divergence time, yet the decrease was not as severe as for the DeepGoPlus, effectively increasing the difference by which MLP outperformed DeepGoPlus. Although less severe, this same trend was also observed between protein-centric performance of the MLP and the Frequency PSI-BLAST method (Figure 5b). For term-centric performance, the difference in performance became smaller with evolutionary distance but stabilizes beyond the Chordata phylum.

We observed deviating behavior in Mouse, Yeast and A. thaliana as their MLP performance did not follow the trend of “increased divergence time, decreased performance.” As the behavior of Mouse might be caused by splitting the data into a train, validation, and test set, we recreated our MLP experiments with Human as the training species as a control experiment (Figure S.5A). We observed a similar trend as before but this time the performance in Human, Yeast and A. thaliana was deviating, indicating that the splitting of the training species into a train, validation and test set was responsible (Figure S.5B and C).

Evaluating using protein-centric semantic distance^39^ confirmed the superiority of the SeqVec-based MLP over Frequency PSI-BLAST and DeepGOPLus (Table S.5). In addition, recent work showed that several wrongful GO annotations exist with the evidence code “IBA.”^40^ However, in our dataset, removing phylogenetic annotations had a very small effect on the results (Supplementary Material subsection 2.2, Figure S.6).

Similar to Littmann et al^41^ we observed a very strong positive correlation between protein-centric SeqVec-based molecular function prediction performance and average sequence identity per species (Spearman correlation: 0.96, *P*-value: 4.5e-05) (Figure 5d), although this trend breaks for proteins with less than 30% identity (Supplementary Material subsection 2.3, Figure S.5D and E). The deviating performance of Yeast could partially be explained by the observed correlation, as Yeast had the lowest (but very similar to A. thaliana) average sequence identity to the training set (Figure S.7).

Finally, it must be noted that besides the quality of the molecular function prediction method, its coverage (ie, the fraction of protein for which at least one prediction was made) is a second most important characteristic. We observed that the coverage of the classifiers trained using SeqVec embeddings as well as for the baseline Frequency PSI-BLAST and DeepGoPlus was 1.0 in all experiments (Figures 5b and S.5C). For the SeqVec-based predictions, 50% to 80% of the proteins were assigned a term of depth at least 4 and these percentages decreased for increasing term depth (Figure S.8B).

Overall, the results reveal the ability of SeqVec-based molecular function prediction to extract information from one well-annotated training species for predictions in various other eukaryotic species.

### Not-evaluated species-specific GO terms contribute only a few annotations

As cross-species molecular function prediction inevitably limits the number of GO terms that can be predicted in test species, we assessed to what extent this affects the integrity of SeqVec-based molecular function prediction. Specifically, a protein has a certain number of real annotations which are all the annotations present in the species datasets, including the non-evaluated GO terms. Given that we only evaluated overlapping GO terms between training and test species, we calculated the percentage of these real annotations that we were able to predict as a quantity for missed predictions.

In all species, we observed a wide distribution of the predicted percentage of real annotations per protein (Figure S.8A). The distributions were heavily tailed to the high percentages for all test species except the Mouse and Yeast, which previously also showed lower performances. Interestingly, with increasing divergence time, the average percentage of predicted real annotations remained fairly constant, whereas the percentage of real GO terms evaluated decreased with increasing divergence time (Table S.4). We hypothesized this observation might be indicating that the filtered-out GO terms were rare terms with few annotated proteins, and hence excluding them from evaluation had only minor influence on the percentage of true predicted GO terms.

To test this, we calculated the Resnik information content (IC)^42^ of each GO term per species. A high IC indicated a rare term in a GO corpus, and a low IC a common term. Indeed, the average IC value of not-evaluated GO terms was high for all species, indicating that the not-evaluated GO terms represent rare molecular functions among the proteins of the test species (Table S.7).

### SeqVec-based molecular function prediction reveals specific types of molecular functions executed by conserved proteins across different species

From the characterization of SeqVec-based molecular function prediction, we know that performance positively correlates with an increased domain, family or superfamily similarity between proteins. We figured that we can exploit this property to identify types of molecular functions executed by more conserved proteins. Specifically, if performance remains constant with increasing divergence time, it would indicate that the proteins are more conserved. Hence, we compared the performance of each GO category between all evaluated species.

**Figure 6.**
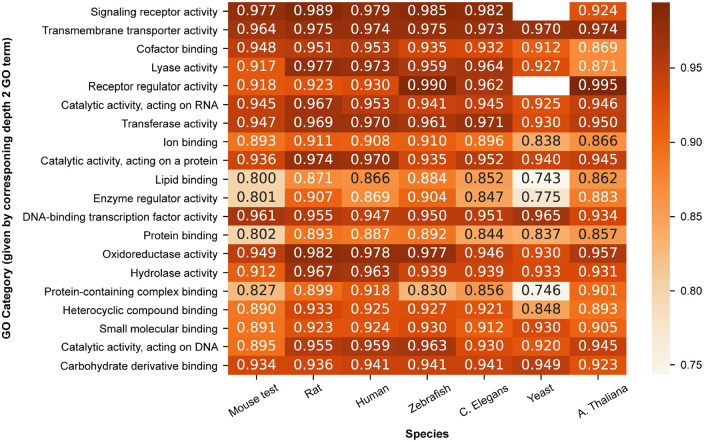
Median term-centric performance (ROCAUC) per GO category per species of the MLP classifier trained using SeqVec protein-level embeddings on the Mouse training dataset. A missing number indicates that a certain GO category was not present among the evaluated proteins.

We observed that indeed some molecular functions indicated by their GO category could be predicted with constant performance across all species, such as “transmembrane transporter activity” (Figure 6). Additionally, we observed GO categories with constant performance among the mammal species and decreased performance in the other species such as “hydrolase activity” and “catalytic activity, acting on a protein,” indicating that these categories of functions might be more conserved among mammals.

As we ordered the GO categories the same as in Figure 4 (best to worst performance), it was interesting to observe that GO categories did not perform similar in the cross-species experiments (ie, they do not have the same order of performance in the different species). Note, the order was previously determined using the SwissProt dataset (which has maximum 30% pairwise sequence identity), so this dataset disregarded many proteins of certain GO categories with high sequence similarity. Given the discrepancies in order and no restrictions on sequence similarity for the cross-species experiments, these results indicate that in reality a large number of similar proteins likely exist for these categories. Overall, these results reveal a possible application for SeqVec-based molecular function prediction in which conserved protein functions could be identified.

### Protein functions from the GO categories biological process and cellular component can also effectively be predicted cross-species

To further assess the potential of cross-species SeqVec based protein function prediction, we tried to predict GO terms from the GO categories Biological Process (BP) and Cellular Component (CC) using the same methodology as before. Again using Mouse as the training species, we created BP and CC datasets with protein annotations for the test species (the number of selected proteins and GO terms can be found in Table S.8).

**Figure 7.**
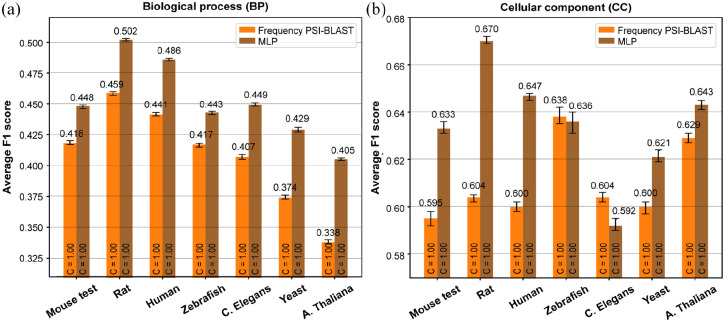
The average protein-centric F1 performance over all the proteins per species for the MLP classifier (brown) for (a) biological process GO terms and (b) cellular component GO terms. Performance is compared to baseline Frequency PSI-BLAST (orange). The coverage C is shown inside the bars. Errorbars denote 95% confidence intervals estimated using 100 bootstraps.

For BP, we observed the same trend as before as the absolute performance of the MLP decreased with increasing divergence time (Figures 7A and S.9A). Again, the decrease was not as severe as for the Frequency PSI-BLAST method, effectively increasing the difference by which MLP outperformed Frequency PSI-BLAST. The term-centric performance of CC also displays this trend while its protein-centric performance does not (Figures 7B and S.9B). Here, performance initially decreases with increasing divergence time, but increases again for Yeast and A. Thaliana. Nevertheless, the MLP outperforms Frequency PSI-BLAST in all species except Zebrafish and C. Elegans.

Although no further experiments were done on the prediction of biological process and cellular component GO terms, these results further support the potential for SeqVec-based protein function prediction in practical applications.

## Discussion

### Performance of SeqVec-based molecular function prediction models dominated by the level of conserved proteins

Protein-level embeddings from the SeqVec model are effective tools in the task of molecular function prediction, reaching competitive performance to many state-of-the-art sequence-based molecular function prediction methods.^24^ Here, we shed light on the “black box’ of SeqVec performance and built upon previous work by characterizing the performance of a simple LR classifier trained using SeqVec embeddings.

Fundamental to the GO hierarchy, GO terms close to the root annotate a wide variety of proteins compared to more specific divergent functions lower in the GO hierarchy. Our findings suggest that inherent to this GO hierarchy, GO terms closer to the root can generally be predicted with lower term-centric performance than GO terms far from the root, despite having more positive examples. This implies that the performance of SeqVec-based molecular function prediction is sensitive to having access to a training set containing proteins roughly similar on a large functional scale, yet still distinct on a smaller scale. A possible countermeasure is offered by “projected predictions” to correct predicted probabilities to respect the GO hierarchy.^15^ Specifically, the probability of a protein being annotated with a term close to the root (eg, “binding”) should never be lower than the probability of being annotated with a child term of it (eg, “DNA binding”). In theory, this could improve the predictions of GO terms close to the root, although it will be less effective for GO terms with many false positives that likely already have high predicted probability scores.

Contrary to our expectation, we also observed a positive correlation between protein-centric performance and protein length. To explain the observed behavior, we suspected that the length of the protein is somehow encoded in SeqVec embeddings. Specifically, as long proteins were relatively scarce in our dataset, they could be easier to predict by having similar embeddings to other long proteins with similar function in the training set. Indeed, we showed that protein length can effectively be predicted from SeqVec embeddings, thereby presenting itself as a protein characteristic that counters the expected decrease in performance for longer proteins. Interestingly, this relevant information on protein length is present in protein-level embeddings, even though they are obtained by taking the mean over amino acid embeddings. This is in line with results from NLP, where sentence embeddings have been shown to be predictive of sentence length, even by averaging the embeddings of all words in a sentence.^43^

As we observed large differences in performance between GO terms with the same depth, we hypothesized that some molecular functions are somehow easier to predict than others. We investigated the possible influence of differences in the domain similarity between annotated proteins on term-centric performance as we hypothesized that GO terms with diverse proteins might be harder to predict. Indeed, the term-centric performance for GO terms with many annotated proteins from the same family is generally better. Additionally, we showed that having multiple protein domains per protein does not interfere with SeqVec’s ability to model protein families. Hence, the SeqVec model is capable of modeling multiple functionalities of proteins in one embedding. Given that SeqVec is trained using biLSTM layers, this observation might indicate that SeqVec might be able to recognize protein domains in protein sequences potentially revealing the underlying mechanism by which it is capable of modeling multiple functionalities of proteins. It would be interesting to follow this up. Moreover, we showed that having a higher prevalence of the shared superfamily among the remaining protein population lowered term-centric performance. The latter observation is in line with our previous notion that SeqVec-based molecular function prediction performance suffers from having to predict proteins with a similar function at the broad scale yet a distinct specific function. Overall, these observations hinted that some molecular functions are executed by a wider spectrum of proteins, thereby decreasing the predictive power of SeqVec-based molecular function prediction. Of note, we were unable to consider all test proteins in the experiments on domain, family and superfamily similarity as such annotations were sparse. Nevertheless, we do not expect large differences in the observed findings if all annotations would be available.

Using these insights, we identified specific groups of molecular functions executed by more similar proteins in terms of their domains and families. The higher domain, family, and superfamily similarity in some GO categories might be explained from an evolutionary perspective: the proteins that execute the molecular functions of well-performing GO categories seem to be more conserved. For instance, our best performing GO category was “signaling receptor activity,”, and the Par proteins, GTPases, kinases, and phosphoinositides that participate in signaling pathways are highly conserved over diverse species.^44^ On the other hand, our worst-performing GO category, “carbohydrate derivative binding,” is executed by proteins with a high degree of complexity and heterogeneity, as reflected by the fact that the proteins are grouped into 45 protein families.^45,46^ Therefore, the performance of SeqVec-based molecular function prediction might be indicative of groups of conserved proteins. For instance, we observed that even within the median performing GO categories some GO terms do have high performance . We speculate that these more specific molecular functions might be executed by a group of conserved proteins, but further research is required to validate this hypothesis. An alternative explanation might be that predictive performance is influenced by similarities in data availability for similar species or by an experimental bias that favors certain types of functionalities for certain species.

### Cross-species SeqVec-based molecular function prediction is possible and offers many fruitful applications in practice

Our work provides a novel evaluation scheme to molecular function prediction based on the annotated protein sequence data of merely one training species. Using the methodology of SeqVec-based molecular function prediction in a transfer learning task, the model effectively extracted information on protein functions from one training species to make predictions available in various other eukaryotic species. This ability to generalize learned protein functions across different kingdoms shows that the trends found by the neural network (both the SeqVec model and the MLP classifier) not only hold for the proteins of the training species but are conserved throughout the eukaryotic domain of life. This was not the case for the supervised-learning-based baseline that failed to generalize to distant species. This confirms our hypothesis that SeqVec-based molecular function prediction is to some extent independent to the context of species and substantiate SeqVec’s capability to model underlying protein principles. It should be noted that all methods were evaluated using the intersection of terms between the training and test species, as species-specific terms not available in the training species cannot be predicted using this approach. We also chose not to include terms unique to the well-annotated training species, as it is not always clear whether these indeed species-specific terms or they are missing due to varying degrees of missing annotations.

We showed that the absolute performance of SeqVec-based molecular function prediction decreased with increasing divergence time, although it was not as severe as for the other methods. We correlated this decrease in performance to a decrease in average sequence identity between the training species and the test species. One explanation to this observation comes from the rule of thumb “increased sequence identity, increased likelihood structural similarity and hence increased likelihood functional similarity” between proteins.^17,34^ We previously noted that SeqVec embeddings of proteins that share a domain or are from the same protein family are likely similar in embedding space, and hence more likely to receive the same molecular function, explaining the observed behavior. However, if functionally similar proteins are indeed similar in embedding space, one might not expect a substantial decrease in performance with increasing divergence time.

However, we note that for proteins in the twilight zone, that is with maximum 30% sequence identity, the correlation between performance and sequence identity disappeared. We attributed this to the fact that the relationship between sequence identity and structural similarity vanishes in the twilight zone, potentially lowering the performance if proteins diverge.^47^ With Yeast and A. thaliana both having a significant proportion of their proteins with a lower than 30% sequence identity, the performance of cross-species function prediction becomes more dependent on the randomness of evolution, that is how many proteins will be divergent by evolution, thereby lowering the performance of SeqVec-based molecular function prediction. This is illustrated by the performance of Yeast, the species with the highest fraction of proteins in the twilight zone and the worst performance, while not being the furthest in divergence time. Overall, this indicates that after some threshold in divergence time from the training species, the molecular function prediction will disobey the observed “increased divergence time, decreased performance” trend. Hence, for high-performance in the test species, the training species should be reasonably evolutionary close. Ideally, one model species per kingdom (Plantae, Fungi, Bacteria) could deal with this problem.

Finally, we also evaluated cross-species SeqVec-based protein function prediction for the GO terms on Cellular Component (CC) and Biological Function (BF), the remaining categories of the Gene Ontology and observed the same behavioral trends in performance as for molecular function predictions.

A possible application for our approach is in cases where limited annotated protein training data is available per taxonomic kingdom. For instance, the UniProtKB/SwissProt knowledgebase has started the Plant Proteome Annotation Program (PPAP) in 2009 to get more annotations on 2 model plant species, Arabidopsis thaliana and Oryza sativa, which could function as the training species in the plant kingdom.^48^ Another application arises with the discoveries of novel protein functions. A recent study on the proteomes of 100 species has identified many highly expressed proteins without any functional annotation or sequence homology to proteins with known annotations.^3^ It is proposed that the exploration of this dark proteome could reveal essential functions for the species which could be of biological or biotechnological interest and a SeqVec-based model might be useful at transferring to many species any novel functions that are experimentally identified functions in 1 species. All in all, we present a novel, data-undemanding protein function prediction evaluation scheme that relies on the availability of merely one adequately annotated model species per evolutionary kingdom and uses the methodology of SeqVec-based molecular function prediction.

## Materials and Methods

### Datasets: SwissProt and cross-species

#### SwissProt dataset

To characterize and improve the performance of SeqVec-based molecular function prediction, we used the SwissProt dataset from previous work.^24^ In short, this dataset contained labeled protein sequence data from the SwissProt database for a selection of proteins with a sequence length in the range [40, 1000]. Every protein had at least one functional GO term annotation from the Molecular Function Ontology (MFO) obtained by non-computational means, that is with one of the following evidence codes: “EXP,”, “IDA,” “IPI,” “IMP,” “IGI,” “IEP,” “HTP,” “HDA,” “HMP,” “HGI,” “HEP,” “IBA,” “IBD,” “IKR,” “IRD,” “IC,” “TAS.”. Data on the annotations of 441 GO terms with at least 40 positive examples in the training set and at least 5 positive examples in the validation and test set was available. The dataset contained 63994 training, 8004 validation, and 3530 test proteins with at most 95% sequence identity to each other. Proteins in the test set had the additional constrain of at most 30% sequence identity to each other and proteins in the training set.

#### InterPro annotations for SwissProt dataset

We retrieved all available domain, family, and superfamily annotations from the InterPro database. We were able to obtain at least 1 domain, family or superfamily annotation for 1631, 2210, or 1687 out of the 3530 test proteins, respectively. To prevent calculating statistics over just 1 or 2 annotated proteins per GO term, we considered only GO terms with at least 50% of its functionally annotated proteins also having InterPro annotations. As a result, we evaluated 278, 355, or 277 out of the 441 GO terms for the domain, family or superfamily similarity, respectively.

#### Cross-species datasets

We tested the ability to transfer knowledge on molecular function between different species using data from 7 model species: Mus musculus (Mouse), Rattus norvegicus (Rat), Homo sapiens (Human), Danio rerio (Zebrafish), Caenorhabditis elegans (C. elegans), Saccharomyces cerevisiae (Yeast), and Arabidopsis thaliana (A. thaliana). Independent for each of the model species, we retrieved data on the sequence and MFO of proteins from the Swiss-Prot Database, only including proteins with at least one MFO annotation obtained using non-computational means. We retrieved gene counts from the Uniprot reference proteomes.^49^ Since mouse was selected as the training species, the mouse data was split into a train, validation and test set using a stratified multi-label split to preserve as many overlapping GO terms as possible between them.^50^ This resulted in 8977 mouse training, 1801 mouse validation, and 1790 mouse test proteins (ratio of $\frac{5}{7}:\frac{1}{7}:\frac{1}{7}$ respectively). An overview of the taxonomic classification, the amount of selected proteins and gene coverage per species is provided in Table S.4A.

### Amino acid-level and protein-level embeddings

We represented amino acids in the form of SeqVec embeddings.^25^ For every amino acid n in the protein sequence, we extracted the d= 1024-dimensional embeddings ( $w_{n}^{1},w_{n}^{2},w_{n}^{3}$ ) $\in {\mathbb{R}}^{d}$ from the 3 layers of the SeqVec model (Figure 1b). As proposed by Heinzinger et al,^25^ we summed these 3 embeddings component-wise using:

(1) $$w_{n}=w_{n}^{1}+w_{n}^{2}+w_{n}^{3}$$

to obtain an amino acid-level embedding $w_{n}\in {\mathbb{R}}^{d}$ .

Using the amino acid-level embeddings, we represented protein sequences with protein-level embeddings.^25^ For a protein of length M , we calculated the protein-level embedding as the component-wise mean over the sequence of amino acid-level embeddings $\left(w_{1},\ldots ,w_{M}\right)$ . Specifically, for every protein we obtained the concise matrix $W=\left[w_{1},\ldots ,w_{M}\right]\in {\mathbb{R}}^{d\times M}$ and calculated the vector $v_{1}(W)$ in ${\mathbb{R}}^{d}$ whose d components were the component-wise arithmetic mean using:

(2) $$v_{1}\left(W\right)=\frac{w_{1}+\cdots +w_{M}}{M}$$

This operation summarized the amino acid sequence of variable length M into a fixed-sized vector $v_{1}(W)$ (Figure 1c). Each of 1024 protein-level features was standardized to 0 mean and unit variance using the training set.

### Molecular function prediction models

#### Models for the SwissProt dataset

We characterized the performance of SeqVec-based molecular function prediction using a Logistic Regression (LR) classifier trained using the protein-level embeddings. The trained classifier predicted for each test protein the probability ∈ [0, 1] of being annotated with a certain GO term.

We trained an independent LR for every GO term using L2 regularization and Stochastic Gradient Descent (SGD) to accelerate the training process. To tune the penalty coefficient λ , we tested the values $10^{x}$ with x ∈ [2,1,0,-1,-2,-3] using the SwissProt validation set. The optimal value was determined jointly over all the 441 GO terms by the highest average ROCAUC score (term-centric evaluation).

#### Model for the cross-species datasets

For the cross-species experiments, we trained an MLP with 1 hidden layer with 512 nodes followed by a ReLu activation function. We applied a dropout to the hidden layer of 30% to prevent overfitting.^51^ The input layer contained a number of nodes equal to the dimension of the input protein-level embeddings, that is 1024. The output layer contained nodes for all the 4086 GO terms in the mouse training set, followed by a Sigmoid activation function, ensuring the MLP outputs are in the range [0, 1]. We trained the MLP in a mini-batch mode (size 64) for 100 epochs using the binary cross entropy averaged over all the GO terms as a loss function. We used the Adam optimizer^52^ for parameter updating at an initial learning rate of $5\cdot 10^{-4}$ that was reduced by a factor of 10 whenever the validation loss did not improve for 5 consecutive epochs. To obtain the optimally trained MLP model, we selected an independent model for term-centric evaluation and protein-centric evaluation determined by the highest validation performance using the Mouse validation set. Additionally, after the MLP predicted the probabilities of GO term annotations, we propagated them to respect the GO hierarchy. Specifically, each parent term in the GO hierarchy received the highest probability score among its child terms, if and only if this score was higher than its own predicted probability score. This GO hierarchy correction step was not done for the experiments using the SwissProt dataset.

### Protein length prediction model

To access if protein-level embeddings modeled protein length, we trained a LR classifier to predict protein length. We used one-hot encoding to model protein length in bins. The LR was implemented as described above. To tune the penalty coefficient λ , we tested the values $10^{x}$ with x ∈ (2,1,0,−1,−2,−3, −4, −5) using the SwissProt validation set. The optimal value was determined jointly over all the ten protein length intervals by the highest average ROCAUC score (term-centric evaluation).

### Baseline method Frequency PSI-BLAST

We used PSI-BLAST with 3 iterations as a baseline method in the cross-species experiments.^16,53^ This baseline showed to be most suitable for the purpose of this paper (Supplementary Material subsection 2.1). We considered all PSI-BLAST hits to the target protein to obtain predicted probability scores for the GO terms as suggested by Radivojac et al^15^ In brief, we annotated each protein with all the GO terms present among all the PSI-BLAST hits. The predicted probability given to each annotation was the frequency of that term among all the PSI-BLAST protein hits, that is the number of (PSI-)BLAST hits annotated to the GO term divided by total number of hits.

### DeepGOPlus

We trained DeepGOPlus^54^ from scratch using only our mouse training set. DeepGOPlus uses a convolutional neural network with one-hot encoded amino acids as input and calculates a final score for a protein-GO term pair using the weighted average of the posterior probabilities of the network and BLAST scores. We use the hyperparameters (learning rate, number of hidden layers, size of convolutional filters, and weights to combine the posterior probabilities) that were reported as optimal for molecular function prediction by the authors.^54^ We used our mouse validation set to decide on the optimal epoch to stop training, using early stopping as recommended, and to find the threshold of posterior probabilities that maximizes the F1 score.

### Performance evaluation

We evaluated all models using the protein-centric F1 score and semantic distance^39^ and term centric ROCAUC, whose definitions can be found in the supplementary material section 3. We estimated 95% confidence intervals using bootstrapping. We obtained a stratified resampled set from the test set with size equal to the original test set. Subsequently, we calculated the evaluation metrics on the resampled set, repeating this process 100 times using a distinct random state. We executed every bootstrapping process on the different datasets with the same random states to enable comparison between them.

#### GO term selection for cross-species evaluation of models

The cross-species datasets differed in the number of unique GO terms present among the selected proteins. To deal with these differences, we evaluated only GO terms overlapping between the training species (Mouse) and the test species in case of protein-centric evaluation. For term-centric evaluation we had additional criteria as the proper calculation of ROCAUC scores needs enough positive examples for each GO term. To this end, we selected GO terms with at least 5 annotated proteins in the mouse training set and at least 3 annotated proteins in the mouse validation, mouse test and the test species test sets. There were 1530 unique GO terms in the mouse training set with ≥5 annotated proteins. Again, we only evaluated GO terms from the test species overlapping with this selection. An overview of the amount of GO terms per species and evaluation metric is provided in Table S.2B.

## Supplemental Material

sj-pdf-1-evb-10.1177_11769343211062608 – Supplemental material for The Power of Universal Contextualized Protein Embeddings in Cross-species Protein Function Prediction Click here for additional data file. Supplemental material, sj-pdf-1-evb-10.1177_11769343211062608 for The Power of Universal Contextualized Protein Embeddings in Cross-species Protein Function Prediction by Irene van den Bent, Stavros Makrodimitris and Marcel Reinders in Evolutionary Bioinformatics

sj-pdf-2-evb-10.1177_11769343211062608 – Supplemental material for The Power of Universal Contextualized Protein Embeddings in Cross-species Protein Function Prediction Click here for additional data file. Supplemental material, sj-pdf-2-evb-10.1177_11769343211062608 for The Power of Universal Contextualized Protein Embeddings in Cross-species Protein Function Prediction by Irene van den Bent, Stavros Makrodimitris and Marcel Reinders in Evolutionary Bioinformatics
