# Supplementary material for: The Power of Universal Contextualized Protein Embeddings in Cross-species Protein Function Prediction
Source: Evol Bioinform Online. 2021 Dec 3;17:11769343211062608. doi: 10.1177/11769343211062608 (PMC8647222; doi:10.1177/11769343211062608)
Supplement: sj-pdf-1-evb-10.1177_11769343211062608 – Supplemental material for The Power of Universal Contextualized Protein Embeddings in Cross-species Protein Function Prediction [file sj-pdf-1-evb-10.1177_11769343211062608.pdf]

The power of universal contextualised protein embeddings in cross-species protein function prediction

Supplementary Material

Irene van den Bent, Stavros Makrodimitris and Marcel J.T. Reinders [1]

[1]To whom correspondence should be addressed.

This document contains supplemental figures, tables and elaborations on the experiments conducted for the paper "The power of universal contextualised protein embeddings in cross-species protein function prediction". Section 1 concerns the characterization part of the paper, section 2 the cross-species part, and section 3 goes into more detail on the materials and methods.

1 Characterization

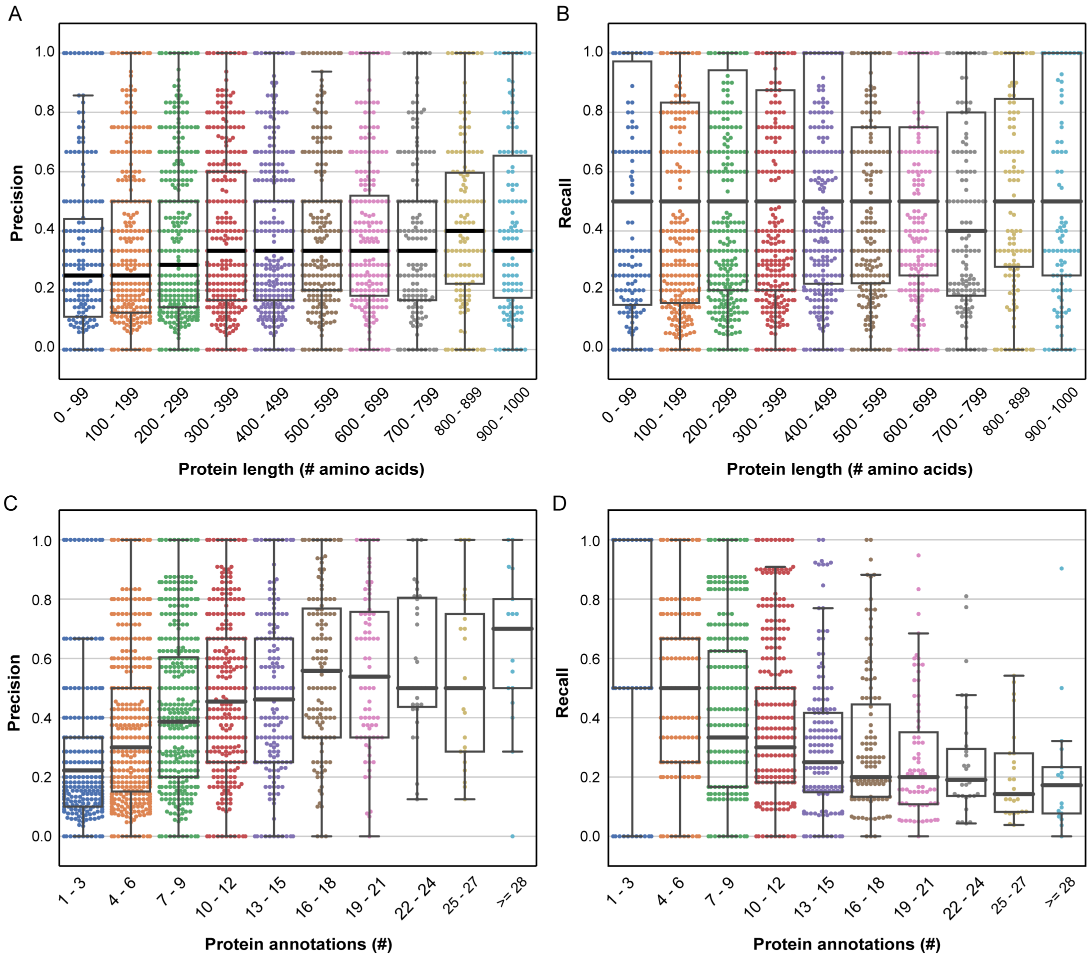

Figure S.1: Protein-centric (A, C) precision and (B, D) recall per protein of LR classifier trained using baseline SeqVec protein-level embeddings on the SwissProt dataset with at most 30% sequence identity in relation to (A, B) protein sequence length and (C, D) the number of protein annotations. The LR was trained to predict GO terms. The box-whiskers plots show the standard IQR, median and whiskers.

1.1 Relationship between term-centric performance, InterPro annotation similarity and term frequency

Differences in class imbalance might be an important confounding factor in the relationship between term-centric performance and the percentage of proteins annotated with a term that share a domain/ family/superfamily annotation (structural similarity of proteins that perform a particular function). Therefore, we set out to establish whether the percentage of proteins that share an interpro annotation is still significantly related to performance if we take the number of positive examples for each term into account. First, there was a very large correlation between the number of positive examples in the training set and in the test set ($\rho = 0.89$, p-value $< 1e-16$), so we only used the training frequency in the following. We used linear regression to model the term-centric ROCAUC (y) as a function of the term absolute frequency in the training set (f) and the structural similarity (s). We log-transformed f and then centered and scaled the three variables of interest. We fitted five different models that capture different possible relationships between the variables:

1. $y = b_0 + \epsilon$ (baseline model with only an intercept)
2. $y = b_1 s + \epsilon$ (model with only structural similarity)
3. $y = b_2 f + \epsilon$ (model with only term frequency)
4. $y = b_1 s + b_2 f + \epsilon$ (model with both independent variables)
5. $y = b_1 s + b_2 f + b_{12} sf + \epsilon$ (model with both independent variables plus an interaction term)

Our aim was to determine whether structural similarity gives any additional information for term-centric performance if we already know the term frequency. If it does not, we expect the third model to be the top-performing, while if it does then either the fourth or the fifth model or both will outperform the third model. For all models we assumed that $\epsilon \sim N(0, \sigma^2)$ (standard linear regression) and used the standard normal distribution as prior for b_0, b_1, b_2, b_{12} and the exponential distribution with $\lambda = 1$ as a prior for σ. We compared the models using leave-one-out log likelihood estimated using Paretto smoothing, which also takes into account differences in overfitting risk due to differences in the number of parameters [4]. Results are shown in Table S.1.

Domain

MODEL	RANK	-LL	δLL	δLLstd error
2	1	735.2	0	0
4	2	737.1	1.9	0.64
5	3	738.5	3.3	0.71
3	4	775.3	40.0	11.4
1	5	793.5	58.3	13.3

Family

MODEL	RANK	-LL	δLL	δLLstd error
5	1	972.1	0	0
2	2	977.9	5.8	5.21
4	3	978.9	6.9	4.85
3	4	1001.8	29.7	10.67
1	5	1011.6	39.5	12.01

Superfamily

MODEL	RANK	-LL	δLL	δLLstd error
5	1	756.3	0	0
4	2	759.3	2.9	3.05
3	3	764.9	8.5	7.48
2	4	768.7	12.4	8.23
1	5	790.7	34.4	12.56

Table S.1: Comparison of the five regression models at predicting the term-centric performance of each GO term when s is defined as similarity of domains (top), family (middle) and superfamily (bottom).

We found that the model with only domain similarity (model 2) is ranked the highest, meaning that term frequency is not affecting this association. The posterior mean of b_2 in model 2 is $+0.43 \pm 0.05$,

meaning that an increase of 1 standard deviation in domain similarity leads to an expected increase of about 0.43 standard deviations in ROCAUC. The relationship for family similarity is more complicated, but we see that all three models that include s outperform the model that only uses term frequency. In addition, the coefficient of s is reliably larger than 0 in all these models:

Model 5, $b_1 = 0.45 \pm 0.08, b_{12} = 0.1 \pm 0.05$

Model 2, $b_1 = 0.30 \pm 0.05$

Model 4, $b_1 = 0.32 \pm 0.06$

These results show that higher percentage of proteins with a shared family within a GO term is indeed associated with increased term-centric performance, even after correcting for term frequency. For the superfamily case, the two models that include both GO term properties are best at describing the variation of ROCAUC, with the interaction model being slightly better. In both cases, the coefficient of s was reliably positive (0.15 ± 0.07 for model 5 and 0.18 ± 0.06 for model 4), showing that again the superfamily similarity is an independent predictor of performance.

If we group the terms by GO category and repeat the analysis we get the results of Table S.2:

Domain

MODEL	RANK	-LL	δLL	δLLstd error
2	0	49.6	0	0
5	1	52.9	3.2	1.67
4	2	53.4	3.7	2.36
1	3	61.1	11.5	5.51
3	4	61.5	11.9	4.72

Family

MODEL	RANK	-LL	δLL	δLLstd error
5	0	53.4	0	0
2	1	60.4	7.0	6.17
1	2	63.8	10.4	5.28
4	3	63.9	10.6	7.52
3	4	64.8	11.4	6.02

Superfamily

MODEL	RANK	-LL	δLL	δLLstd error
1	0	60.8	0	0
3	1	62.1	1.3	2.47
2	2	62.4	1.6	4.60
4	3	65.5	4.8	5.18
5	4	68.6	7.8	4.25

Table S.2: Comparison of the five regression models at predicting the mean term-centric performance of each GO category when s is defined as similarity of domains (top), family (middle) and superfamily (bottom).

For domains, as in the ungrouped case, domain similarity without including term frequency is the best predictor of performance and has a reliably positive coefficient with posterior mean and variance equal to 0.62 and 0.18 respectively. For families, the term frequency is now an important confounder as a model that contains both variables and an interaction term is the most predictive of performance. In this model, the positive association of s is confirmed by the reliably positive coefficient it has with posterior mean equal to 0.47, posterior variance equal to 0.17 and $p(b_1 > 0) = 0.998$. For the superfamilies, we had found no significant association with performance on the aggregate dataset and this is confirmed by the inability of all models to significantly outperform the baseline model with only an intercept.

We obtained very similar model rankings and weights when replacing our leave-one-out log likelihood with the Widely Applicable Information Criterion [4]. Finally, to ensure that these findings do not rely on the choice of prior distributions, we repeated the experiments by changing the prior of the weights to $N(0, 10)$ and/or the prior of σ to an exponential with $\lambda = 0.2$ and obtained nearly identical posterior distributions (data omitted for brevity).

Together these results show that the associations we find between similarity of interpro features and term-centric performance still hold even when correcting for the class imbalance across terms.

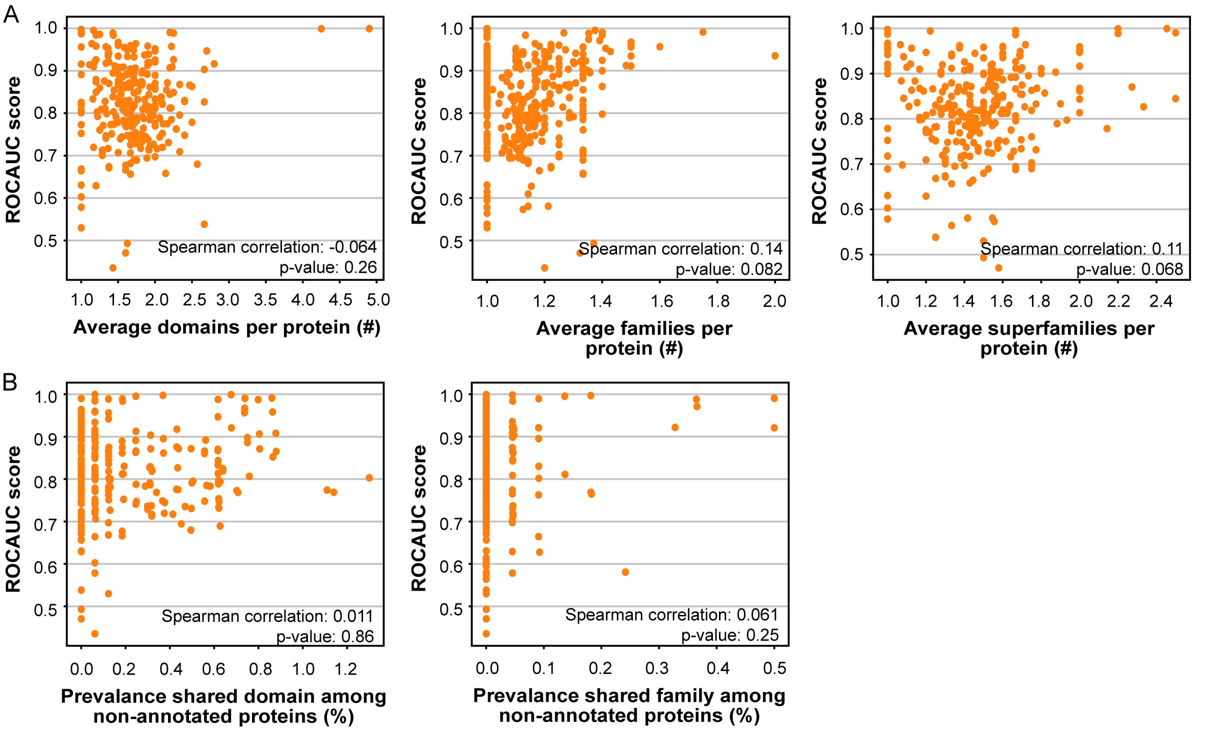

Figure S.2: Term-centric performance (ROCAUC) per GO term of LR classifier trained using baseline SeqVec protein-level embeddings on the SwissProt dataset with at most 30% sequence identity in relation to (A) average number of domain, family or superfamily annotations per protein and (B) prevalence of the shared domain or superfamily among the remaining non-annotated proteins. The LR was trained to predict GO terms. The Spearman correlations and corresponding p-values between term-centric performance and the structural similarity measures are shown. Note that not all GO terms from the SwissProt dataset were evaluated due to a lack of structural annotations (278, 355 or 277 out of the 441 GO terms for the domain, family or superfamily similarity, respectively).

GO Category	Child GO terms (#)	Median performance (ROCAUC)	Average annotated proteins with shared domain (%)	Average annotated proteins with shared family (%)	Average annotated proteins with shared superfamily (%)	Average prevalence shared superfamily among non-annotated proteins (%)
Signaling receptor activity	7	0.995 (0.000)	66	60	NaN	NaN
Transmembrane transporter activity	69	0.927 (0.003)	61	24	68	0.81
Cofactor binding	8	0.919 (0.004)	31	21	38	0.43
Lyase activity	5	0.891 (0.004)	33	20	32	0.34
Receptor regulator activity	5	0.891 (0.004)	NaN	20	25	0.71
Catalytic activity, acting on RNA	20	0.890 (0.004)	28	21	39	0.93
Transferase activity	61	0.866 (0.006)	47	19	49	0.77
Ion binding	26	0.861 (0.010)	26	14	34	1.0
Catalytic activity, acting on a protein	35	0.861 (0.013)	38	20	38	0.48
Lipid binding	7	0.860 (0.015)	41	27	44	0.56
Enzyme regulator activity	32	0.853 (0.016)	24	36	32	0.01
DNA-binding transcription factor activity	7	0.852 (0.004)	29	35	34	0.27
Protein activity	76	0.838 (0.015)	29	23	33	0.87
Oxireductase activity	21	0.832 (0.006)	40	18	40	0.46
Hydrolase activity	84	0.830 (0.012)	25	21	39	1.0
Protein-containing complex binding	5	0.823 (0.002)	19	18	22	1.8
Organic cyclic compound binding	64	0.794 (0.008)	19	16	36	2.4
Small molecule binding	28	0.746 (0.011)	24	15	47	2.9
Catalytic activity, acting on DNA	10	0.724 (0.007)	32	34	52	2.6
Carbohydrate derivative binding	14	0.716 (0.007)	24	16	54	3.5

Table S.3: Overview of structural similarity measures per GO category. NaN values indicate a lack of data. Parentheses behind the median term-centric performance (ROCAUC) correspond to the standard deviation of the median performance. The average percentage of annotated proteins with a shared domain, family or superfamily and the average prevalence of the shared superfamily among the non-annotated proteins was calculated over all the child terms in the GO category.

2 Cross-species function prediction

A

Species	Kingdom	Phylum	Class	# selected proteins	Coverage of gene counts (%)
Mouse	Animalia	Chordata	Mammalia	12.568	57%
Rat	Animalia	Chordata	Mammalia	6.184	29%
Human	Animalia	Chordata	Mammalia	14.823	72%
Zebrafish	Animalia	Chordata	Actinopterygii	1.883	7%
C. elegans	Animalia	Nematoda	Chromadorea	2.732	14%
Yeast	Fungi	-	-	4.358	72%
A. thaliana	Plantae	-	-	10.888	40%

B

Species	# GO terms	# GO terms evaluated protein-centric (% of terms)	# GO terms evaluated term-centric (% of terms)
Mouse	4.572	-	-
Mouse training	4.086	-	-
Mouse validation	2.215	1.964 (89%)	833 (38%)
Mouse test	2.238	1.966 (88%)	854 (38%)
Rat	3.394	3.556 (90%)	1.468 (43%)
Human	4.684	4.008 (86%)	1.522 (32%)
Zebrafish	1.678	1.582 (94%)	653 (39%)
C. elegans	1.968	1.816 (92%)	787 (40%)
Yeast	2.547	1.955 (77%)	839 (33%)
A. thaliana	2.976	1.977 (66%)	898 (30%)

Table S.4: List of species used in this study. (A) Taxonomic rankings, number of proteins and gene count coverage for each species. Gene count is a measure for the number of protein-coding genes in the genome [3]. (B) The number of GO terms (evaluated) in each species. During protein-centric evaluation GO terms overlapping with the Mouse training set are considered. During term-centric evaluation only GO terms with at least 3 annotated proteins and overlapping with the Mouse training set are considered. The mouse dataset consists of the mouse training, validation and test sets combined.

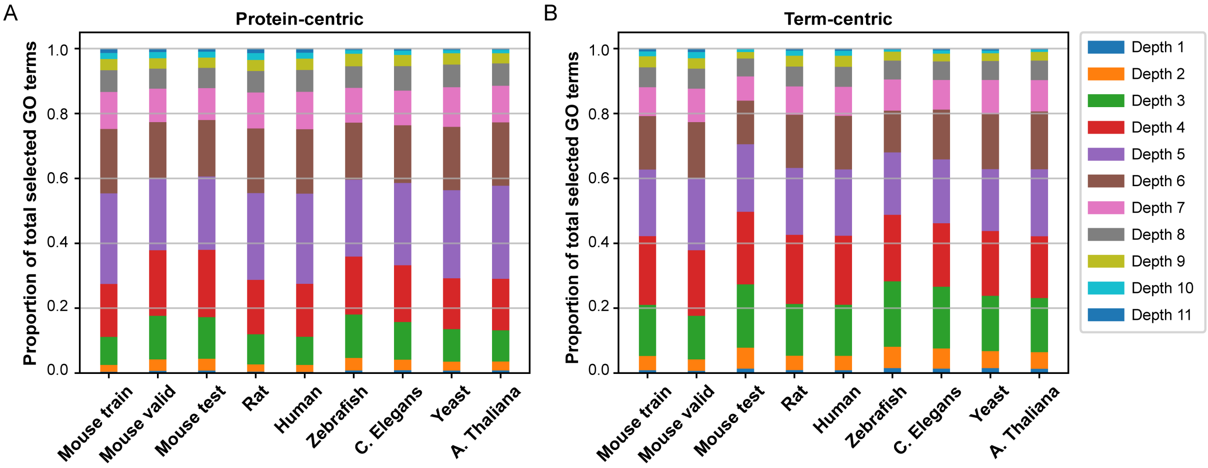

Figure S.3: Distribution of the depth of selected GO terms per species dataset for (A) protein-centric evaluation and (B) term-centric evaluation. Note, in each species a different total number of selected GO terms is present. For at least one species the distribution of GO term depth for protein-centric evaluation was significantly different (Chi-square test p-value: 2.7e-23). This was not the case for the depth distributions of GO terms selected for term-centric evaluation (Chi-square test p-value: 0.20).

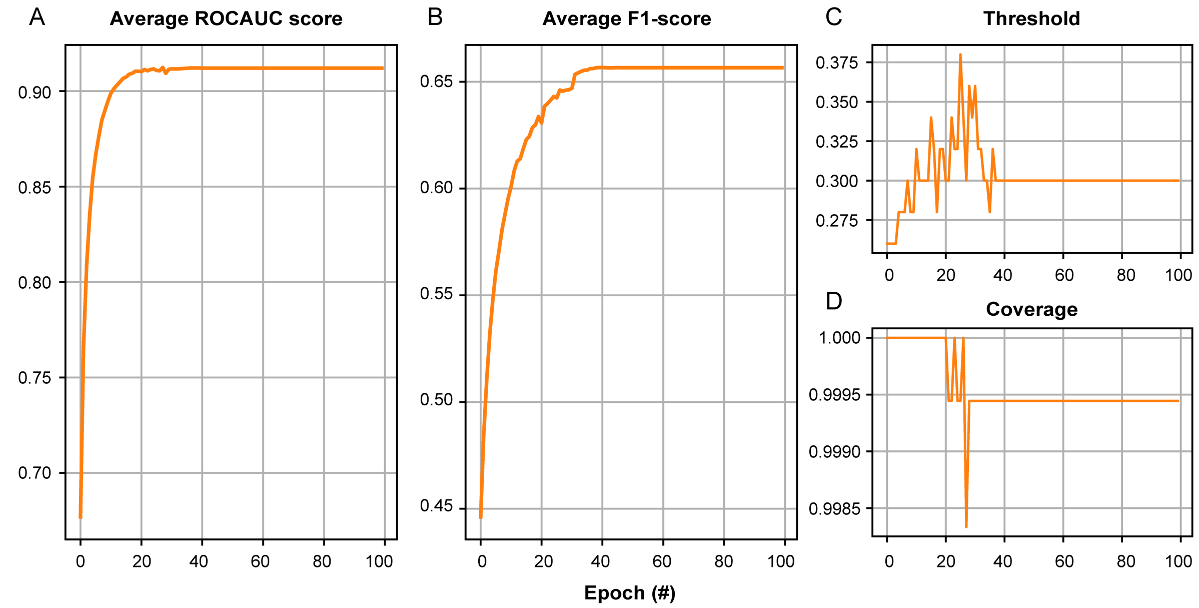

Figure S.4: Average (A) term-centric (ROCAUC) and (B) protein-centric (F1) validation set performance over all the GO terms for MLP classifier trained using baseline embeddings on the Mouse training set. The optimally trained MLP model was selected based on the highest average ROCAUC scores over all the GO terms and the highest F1 score over all the proteins. Note, the MLP was tuned independently for protein-centric and term-centric performance. Each Fmax value was caulated using a certain (C) the threshold on predicted class label probabilities to obtain binary class label predictions. A certain (D) coverage was the result of this threshold. Coverage is the proportion of proteins with at least one predicted GO annotation for the set threshold given the total number of proteins to classify.

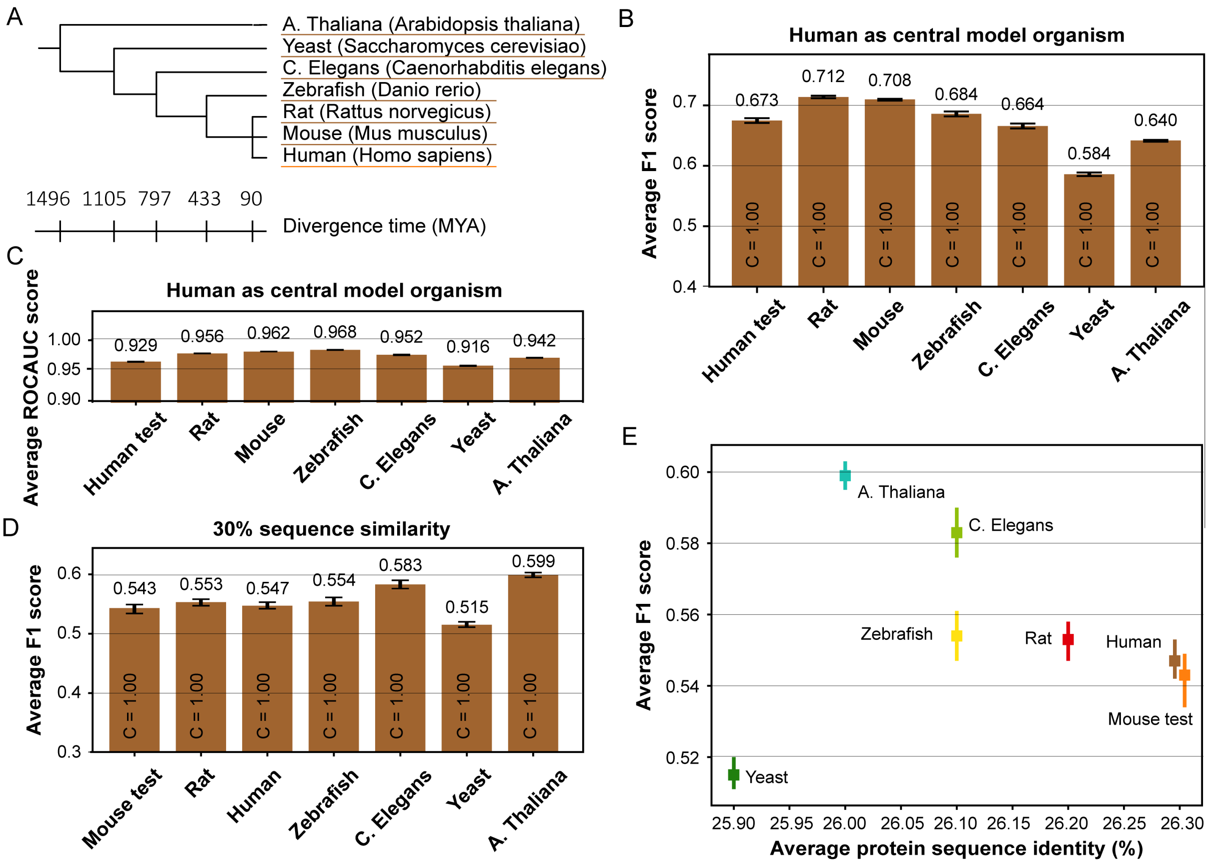

Figure S.5: (A) Phylogenetic tree showing evolutionary relation and divergence time between the train species Human and the other test species, both indicated by colour. Tree produced via the PhyloT tool for phylogenetic tree visualisation and divergence times retrieved using the TimeTree tool. (B) The average protein-centric (F1) performance over all the proteins and (C) average term-centric (ROCAUC) performance over all the GO terms per species of MLP classifier trained using baseline SeqVec protein-level embeddings on the Human training dataset. The MLP was trained to predict GO terms. (D) The average protein-centric (F1) performance over proteins with a maximum 30% sequence identity to the Mouse training set per species of MLP classifier trained using baseline SeqVec protein-level embeddings on the Mouse training dataset. The MLP was trained to predict GO terms. In (B, D) the coverage is shown inside the bars. (E) Average protein-centric performance (F1) over proteins with a maximum 30% sequence identity to the Mouse training set per species of the same MLP classifier in relation to the average protein sequence identity to the Mouse training set. Errorbars denote 95% confidence intervals.

	SeqVec	DeepGOPlus	Frequency PSI-BLAST
Mouse test	**14.23**	18.17	17.58
Rat	**11.43**	14.64	19.91
Human	**10.07**	15.11	15.8
Zebrafish	**8.6**	12.23	11.46
C. elegans	**9.99**	13.74	12.11
yeast	**11.25**	15.77	14.52
A. Thaliana	**9.36**	12.28	12.1

Table S.5: Evaluation of the tested methods based on protein-centric semantic distance on the cross-species dataset. Lower semantic distance signifies better performance. The best performing model for each species is shown in bold.

2.1 Baseline selection

The head-to-head comparison (Table S.6) shows that PSI-BLAST performs better than BLAST except for rat (the closest species to our training species). As far as coverage is concerned, PSI-BLAST's ability to find a hit for many more proteins (i.e. higher coverage) in distant species than BLAST makes it a more relevant baseline. For that purpose, we do not include the BLAST results, since they do not provide a stronger baseline here.

Next, we compare two different PSIBLAST-based options, one based on the top-hit (i.e. maximum sequence identity between the target protein and all the PSI-BLAST hits) and the second based on frequency (i.e. fraction of hits that have a term). Head-to-head comparison demonstrated that the frequency-based PSI BLAST outperformed top-hit-based PSI BLAST in terms of protein-centric F1 for most species, while it had nearly 100% coverage for all species. The performance advantage of this method concerned mostly more distant species. In terms of term-centric ROCAUC, the two methods performed similarly. Given the frequency-based PSIBLAST baseline to have a better coverage and better ability to predict for distant species, which both are of key interest for cross-species predictions, we selected this option as our baseline method.

	F1			COVERAGE			ROCAUC		
	top-hit BLAST	top-hit PSIBLAST	frequency PSIBLAST	top-hit BLAST	top-hit PSIBLAST	frequency PSIBLAST	top-hit BLAST	top-hit PSIBLAST	frequency PSIBLAST
Mouse	0.463	0.488	**0.546**	0.69	0.76	**1**	**0.82**	**0.826**	**0.827**
Rat	**0.727**	0.704	0.575	0.90	0.92	**1**	**0.944**	**0.949**	0.929
Human	**0.649**	**0.644**	0.569	0.87	0.9	**1**	**0.931**	**0.934**	0.916
Zebrafish	0.645	**0.672**	0.579	0.85	0.9	**1**	**0.934**	**0.937**	0.921
C. elegans	0.43	0.501	**0.587**	0.59	0.66	**1**	0.902	**0.911**	**0.913**
Yeast	0.311	0.339	**0.489**	0.42	0.53	**1**	0.812	**0.822**	**0.828**
A. Thaliana	0.331	0.365	**0.557**	0.45	0.57	**1**	0.844	0.849	**0.858**

Table S.6: Comparison of (PSI-)BLAST-based baseline options. Best performance indicated in bold.

2.2 Including phylogeny-based annotations in the cross-species datasets does not alter results

A recent paper by Wei et. al. [5] has demonstrated that phylogeny-based annotations (i.e. with evidence codes 'IBA', 'IBD', 'IKR', 'IRD') are known to have poor accuracy, even lower than fully automated annotations with IEA evidence. Including them as ground truths in our cross-species training set could have a large impact.

Indeed, IBA is a very common evidence code in our dataset, but the other three codes combined comprise less than 0.01% of each species's annotations. We repeated our cross-species experiment excluding all annotations with evidence codes IBA, IBD, IKR, and IRD, following the same procedure regarding training and evaluation as previously. The results, shown in Figure S.6, show that exclusion of these evidence codes has only a minor effect on the final results. The overall pattern of performance across species remains the same (for protein-centric F1 Pearson's ρ=0.990, $p<1e-4$ and for term-centric ROCAUC ρ=0.997, $p<1e-6$, similar values for Spearman's ρ) and the absolute performance values change no more than 4.1% for the F1 score and 1.1% for the term-centric ROCAUC. At the same time, the confidence intervals also remain relatively similar despite the reduction in the number of test proteins.

A possible reason for the small differences is that the large number of IBA misannotations reported by Wei et al. refer to UniProt releases from 2018 and 2019, while we used a 2020 release. As mentioned by Wei et al., their method is used to continuously search for misannotations, so it is possible that a significant portion of them might have been fixed.

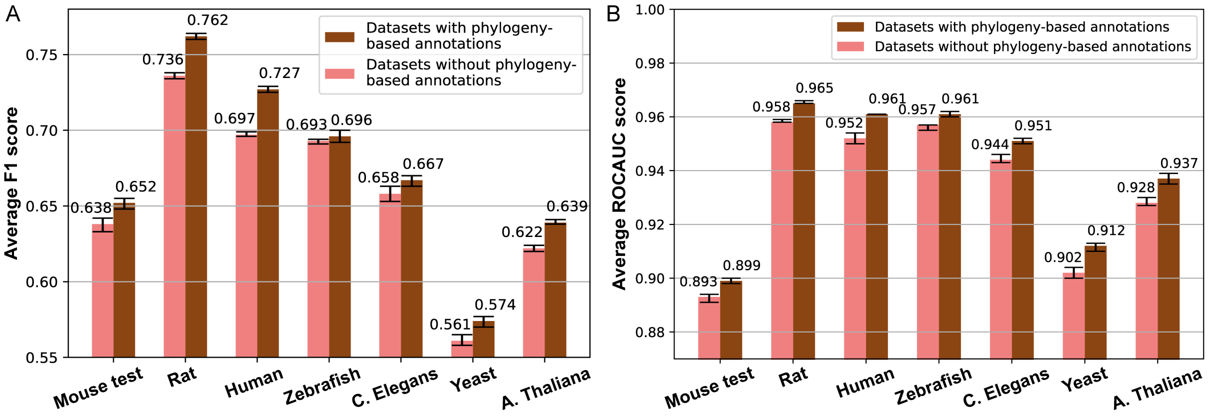

Figure S.6: (A) Protein-centric F1 score and (B) term-centric ROCAUC in the cross species experiment when including (brown) and excluding (pink) phylogeny-based annotations, i.e. with evidence codes IBA, IBD, IKR, and IRD.

2.3 Cross-species protein-centric performance positively correlates with protein sequence identity

Using the BLAST top hit of every protein to find its sequence identity to the training set (the ratio of the number of exact matches to the total alignment length), we observed a very strong positive correlation between protein-centric SeqVec-based molecular function prediction performance and average sequence identity per species (Spearman correlation: 0.96, p-value: 4.5e-05) (Figure 5D).

From the distributions of the sequence identity, we observed that with increasing divergence time the distributions skewed from high sequence identity to low sequence identity (Figure S.7). Theprevious observed deviant performance of Yeast could partially be explained by the observed correlation, as Yeast had the lowest (but very similar to A. thaliana) average sequence identity. The performance of A. thaliana in relation to its average sequence identity was more corresponding with the other test species, indicating that Yeast is likely breaking the trend. On the other hand, proteins in the 'twilight zone' have below 30% sequence identity resulting in limited structural similarity, decreasing the likelihood of conserved protein function [1, 2].

As the amount of sequence identity and the divergence time between species are related, we repeated the MLP experiments while evaluating only proteins from the twilight zone. While we observed a very similar average sequence identity in all species after the sequence identity constraint, the spread in performance remained large (Figure S.5D). Additionally, the species with the highest sequence identity did no longer have the best performance (Figure S.5E). We no longer observed a statistically significant correlation between performance and sequence identity (Spearman correlation: -0.21, p-value: 0.64). These findings illustrate that for proteins in the twilight zone their sequence identity is no longer indicative of performance. As we observed that Yeast had the highest fraction of proteins in the twilight zone, this possibly explains the low Yeast performance (Figure S.7).

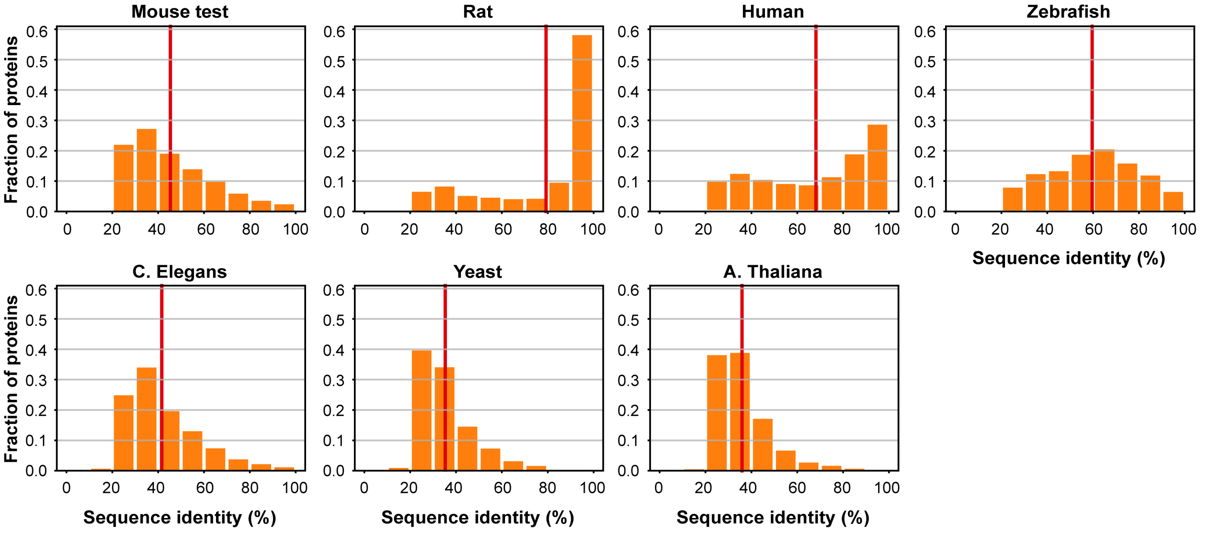

Figure S.7: Distributions of the percentage of sequence identity to the Mouse training set for each protein per species. Bin counts were divided by the total number of proteins in each species datasets to yield fractions of evaluated proteins, aiding cross-species comparison. The vertical red line corresponds to the average protein sequence identity.

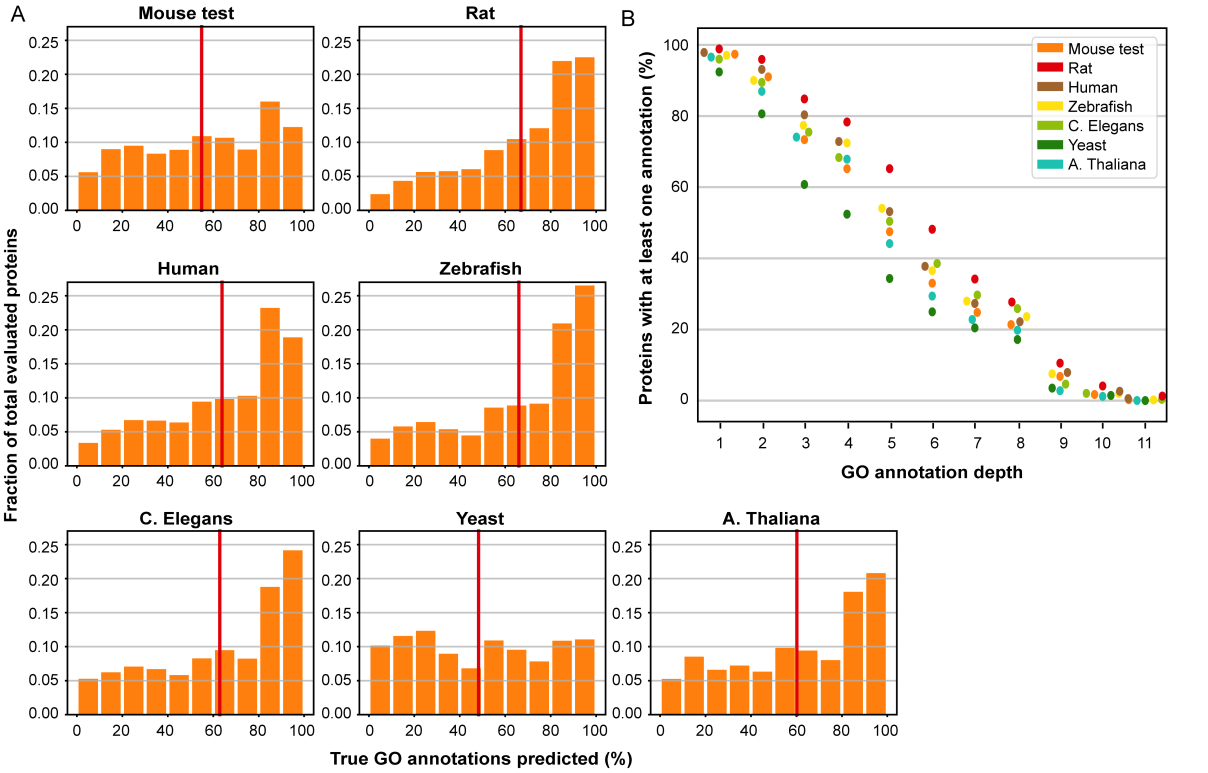

Figure S.8: (A) Distributions of the percentage of predicted real annotations for each protein per species. Real annotations are all the annotations present in the species datasets, including the non-evaluated GO terms. Bin counts were divided by the total number of proteins in each species datasets to yield fractions of evaluated proteins, aiding cross-species comparison. Annotations were predicted by an MLP classifier trained using baseline SeqVec protein-level embeddings using the cross-species datasets. The vertical red line corresponds to the average assigned percentage of true annotations. (B) Percentage of proteins with at least one true positive predicted annotation in relation to the GO term depth. Predictions were made using the same MLP classifier trained using baseline SeqVec protein-level embeddings on the mouse training set. Species are indicated by colour.

Species	Average IC non evaluated GO terms
Mouse validation	10.3 (0.2)
Mouse test	10.3 (0.2)
Rat	11.6 (0.3)
Human	12.2 (0.4)
Zebrafish	10.1 (0.3)
C. elegans	10.5 (0.4)
Yeast	10.8 (0.5)
A. thaliana	11.3 (0.8)

Table S.7: Average Information Content (IC) of the non-evaluated GO terms in protein-centric evaluation per species. Parenthesis indicate standard deviation.

A — Biological Process

Species	# selected proteins	# GO terms	# GO terms evaluated protein-centric (% of terms)	# GO terms evaluated term-centric (% of terms)
Mouse	13.795	15.482	-	-
Mouse training	9.836	14.360	-	-
Mouse validation	1.992	9.194	8.553 (93%)	4.335 (47%)
Mouse test	1.967	9.124	8.528 (93%)	4.400 (48%)
Rat	6.526	13.978	12.991 (93%)	7.023 (50%)
Human	16.067	15.434	13.984 (91%)	7.264 (47%)
Zebrafish	2.204	5.903	5.594 (95%)	2.710 (46%)
C. elegans	2.888	5.127	4.725 (92%)	2.580 (50%)
Yeast	5.122	5.192	4.139 (80%)	2.382 (46%)
A. thaliana	11.257	5.607	4.099 (73%)	2.432 (43%)

B — Cellular Component

Species	# selected proteins	# GO terms	# GO terms evaluated protein-centric (% of terms)	# GO terms evaluated term-centric (% of terms)
Mouse	14.263	1.932	-	-
Mouse training	10.144	1.850	-	-
Mouse validation	2.077	1.867	1.115 (60%)	585 (31%)
Mouse test	2.042	1.815	1.143 (63%)	569 (31%)
Rat	6.655	1.695	1.633 (96%)	881 (52%)
Human	17.107	1.950	1.807 (93%)	947 (49%)
Zebrafish	2.269	879	863 (98%)	443 (50%)
C. elegans	2.944	924	890 (96%)	502 (54%)
Yeast	5.416	1.043	811 (78%)	467 (45%)
A. thaliana	12.096	919	743 (81%)	457 (50%)

Table S.8: The number of (A) biological process and (B) cellular component GO terms (evaluated) in each species. During protein-centric evaluation GO terms overlapping with the Mouse training set are considered. During term-centric evaluation only GO terms with at least 3 annotated proteins and overlapping with the Mouse training set are considered. The mouse dataset consists of the mouse training, validation and test sets combined.

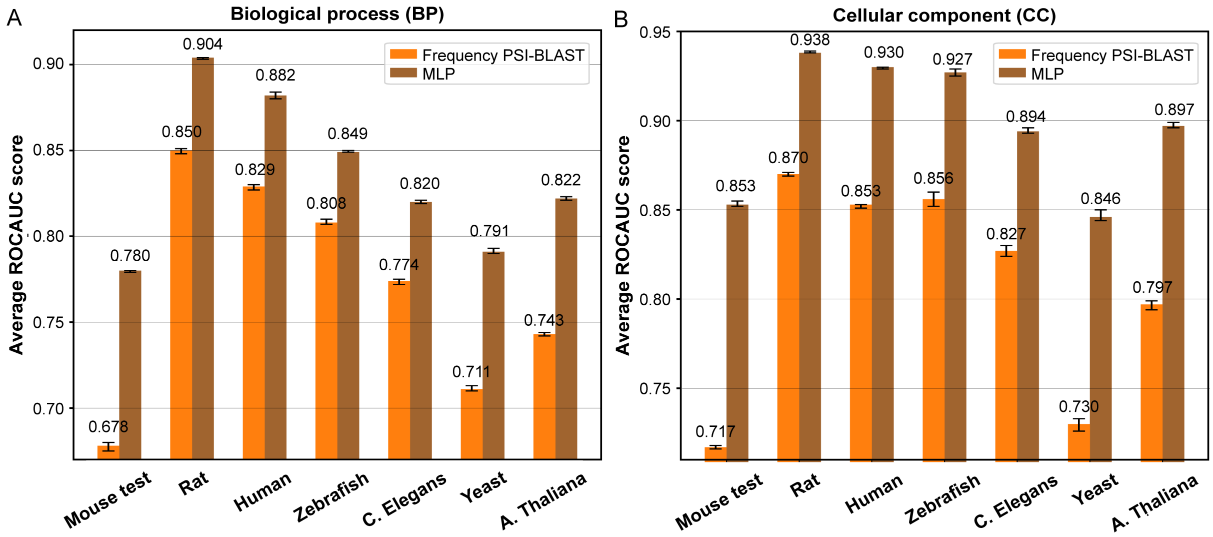

Figure S.9: The average term-centric ROCAUC performance over all the GO terms per species for the MLP classifier (brown) for (A) biological process GO terms and (B) cellular component GO terms. Performance is compared to baseline PSI-BLAST (orange). The coverage C is shown inside the bars. Errorbars denote 95% confidence intervals estimated using 100 bootstraps.

3 Materials and Methods

Protein-centric metrics

For protein-centric evaluation, we calculated the F1-scores of all test proteins. To calculate the F1-scores, we first calculated for some decision threshold $t \in [0, 1]$, which converted the predicted probabilities into binary class labels, the precision $pr_o(t)$ and recall $rc_o(t)$ for every protein o. Using these, we calculated the F1-score $F_{1o}(t)$ for every protein.

To calculate the average F1-score over all the proteins, we first calculated the average precision and recall over all the proteins for a fixed threshold t using:

$$\mathrm{pr}(t) = \frac{1}{U(t)} \cdot \sum_{o=1}^{U(t)} \mathrm{pr}_o(t) \tag{1}$$

and

$$rc(t) = \frac{1}{V} \cdot \sum_{o=1}^{V} rc_o(t) \tag{2}$$

Note that we calculated the precision only over the $U(t)$ proteins for which at least one prediction was made above threshold t and Recall over all the V proteins. Consequently, we obtained the prediction coverage using:

$$C(t) = \frac{U(t)}{V} \tag{3}$$

which represents the fraction of protein with at least one predicted annotation for threshold t, regardless of the prediction being false or true.

Finally, we calculated the average F1-score over all the proteins using:

$$F_1(t) = \frac{2 \cdot \mathrm{pr}(t) \cdot \mathrm{rc}(t)}{\mathrm{pr}(t) + \mathrm{rc}(t)} \tag{4}$$

over all the thresholds t. We calculated the maximum F_1-score over all thresholds using:

$$F_1 = \max_t \{F_1(t)\} \tag{5}$$

To recreate a real case scenario in the cross-species experiments in which no validation set is available for the test species, we determined the threshold t resulting in the highest $F_{\max}$ score on the mouse validation set. Using this fixed threshold, we calculated the F_1-score in the other species. By applying that threshold, we also calculated the semantic distance (SD), defined based on remaining uncertainty (ru) and misinformation (mi), as shown on equations 6-8, where T_o is the set of true annotations of protein o, $P_o(t)$ a model's predicted GO terms for protein o at threshold t and τ indexes the GO terms. The term information content (IC) was calculated by pooling the entire cross-species dataset (training, validation and test sets).

$$ru(t) = \frac{1}{V} \sum_{o=1}^{V} IC(\tau) \cdot I(\tau \in T_o \land \tau \notin P_o(t)) \tag{6}$$

$$mi(t) = \frac{1}{V} \sum_{o=1}^{V} IC(\tau) \cdot I(\tau \notin T_o \land \tau \in P_o(t)) \tag{7}$$

$$SD(t) = \sqrt{ru(t)^2 + mi(t)^2} \tag{8}$$

Term-centric metrics

For term-centric evaluation, we calculated the area under the ROC curve (ROCAUC score) of all GO terms. The average ROCAUC score was obtained by averaging over all the evaluated GO terms.

References

[1] William R Pearson. "An introduction to sequence similarity ("homology") searching". In: *Current protocols in bioinformatics* 42.1 (2013), pp. 3–1.

[2] Burkhard Rost. "Twilight zone of protein sequence alignments". In: *Protein engineering* 12.2 (1999), pp. 85–94.

[3] UniProt Consortium. *Proteomes results.* url=https://www.uniprot.org/proteomes/. 2019.

[4] Aki Vehtari, Andrew Gelman, and Jonah Gabry. "Efficient implementation of leave-one-out cross-validation and WAIC for evaluating fitted Bayesian models". In: *arXiv preprint arXiv:1507.04544* (2015).

[5] Xiaoqiong Wei et al. "Detecting Gene Ontology misannotations using taxon-specific rate ratio comparisons". In: *Bioinformatics* 36.16 (2020), pp. 4383–4388.
